# Thermal Shifts Dominate Single‐Gene Responses to Transcription‐Targeting Antibiotics

**DOI:** 10.1002/advs.77965

**Published:** 2026-09-27

**Authors:** Rahul Jagadeesan, Amir M. Arsh, Vatsala Chauhan, Suchintak Dash, Pooja Ravaria, Andre S. Ribeiro

**Affiliations:** ^1^ Faculty of Medicine and Health Technology Tampere University Tampere Finland; ^2^ Department of Cell‐ and Molecular Biology Uppsala University Uppsala Sweden; ^3^ Kavli Institute of Nanoscience Discovery University of Oxford UK

**Keywords:** antibiotic resistance, antibiotic response, cold‐shock, dual‐stress response, efflux regulation, heat‐shock, temperature

## Abstract

Combined exposure to thermal and antibiotic stresses is ubiquitous. How cells adapt to these concurrent cues at the single‐gene level remains unresolved. Here, we show that in *Escherichia coli*, both cold and heat shock responses dominate antibiotic stress responses across multiple levels of physiology. Using RNA‐seq, we demonstrate that combined exposure produces transcriptomes that are largely indistinguishable from those induced by thermal shifts alone. Flow‐cytometry measurements across intermediate temperatures and increasing ofloxacin concentrations further show that temperature‐like responses are more prevalent outside the 30°C–39°C range. This dominance arises from temperature‐driven changes in ATP levels and nucleoid organization, which in turn modulate DNA gyrase and RNA polymerase engagement with DNA. Combined exposure also induces SoxS‐ and MarA‐mediated efflux systems more strongly than individual stresses. Machine learning analysis identifies CRP and σ^38^ as key gene expression regulators under combined stress. Molecular dynamics simulations show that drug‐target conformational plasticity is altered under combined conditions. Cross‐species simulations across six bacterial species with distinct pathogenic profiles reveal that thermal modulation of antibiotic response is partially conserved across evolutionarily distant species. Together, the results identify temperature as a critical determinant of antibiotic response, suggesting that thermal shifts may facilitate bacterial adaptation to antibiotics.

## Graphical abstract

Thermal shifts reshape bacterial responses to transcription‐targeting antibiotics. In E. coli, temperature can dominate antibiotic‐induced genome‐wide expression, particularly outside 30°C–39°C, through changes in adenosine triphosphate (ATP), nucleoid organization, and drug‐target engagement. Combined stresses also enhance efflux regulation, while cross‐species simulations suggest that thermal modulation of antibiotic responses is partially conserved across bacteria.

## 1 Introduction

Since their earliest divergence, bacteria have evolved complex regulatory programs to survive thermal shifts. In *Escherichia coli*, the heat shock response, orchestrated by σ^32^ [1], induces molecular chaperones and proteases to maintain protein‐folding homeostasis, while also modulating membrane integrity, redox balance, and transient DNA relaxation [2, 3, 4]. Conversely, cold‐shock triggers the production of cold‐shock proteins [5], modifies the transcription initiation kinetics and DNA supercoiling [6, 7], perturbs membrane fluidity and cytoplasmic viscosity [5, 8], and can even promote biofilm formation [9].

Importantly, the physiological consequences of temperature changes depend on their magnitude. In *E. coli*, temperatures within the intermediate range of approximately 30°C–39°C generally produce less pronounced temperature‐dependent responses, whereas conditions outside this range increasingly engage cold‐ or heat‐associated physiological programs. A shift to 25°C represents a moderate temperature downshift, while exposure to 15°C induces a pronounced cold‐shock response followed by an acclimation phase [5]. At higher temperatures, heat‐shock‐associated responses become progressively more prominent above ∼39°C, with exposure to 42°C activating the canonical heat‐shock response [10].

Meanwhile, antibiotics emerged much later in the evolutionary trajectory as microbial secondary metabolites mediating cellular competition and communication [11, 12]. Today, they are indispensable in modern medicine, with the current arsenal broadly divided into bactericidal and bacteriostatic agents [13, 14]. Although their primary modes of action are relatively simple [15, 16, 17], their downstream effects are far more complex, including redox imbalance, radical formation, disrupted signaling cascades, metabolic perturbations, and premature transcription termination [18, 19, 20, 21, 22].

In natural environments, bacteria frequently encounter thermal shifts and antibiotics concurrently. For example, during infections, pathogens may experience febrile host temperatures, together with antibiotic regimes [21]. Ecological settings, such as wastewater treatment, hospital effluents, aquaculture systems, and agricultural soils, can expose bacteria to antibiotic residues across much broader temperature ranges. In such settings, temperatures around 15°C may occur in cold wastewater, temperate aquaculture systems, or winter soils, whereas approximately 25°C–30°C is common in aquaculture and agricultural environments [22, 23]. Temperatures approaching 40°C–2°C are less representative of most host infections but may occur transiently in warm wastewater, tropical surface waters, or open‐air sites experiencing intense solar radiation and seasonal heat waves [22, 23]. Although these contexts differ substantially in both thermal range and antibiotic exposure, responses to thermal and antibiotic stress are most often studied separately, thus limiting our understanding of whether these stresses have either synergistic, antagonistic, or dominant effects.

A recent study by Cruz‐Loya et al. [24] showed that under combined thermal and antibiotic stress, both the optimal growth temperature and the breadth of thermal tolerance shifted in an antibiotic‐dependent manner. Thermal shifts have also been reported to enhance bacterial survival under antibiotic treatment [25, 26]. Conversely, heat‐dependent phase transitions in the outer membrane can modulate the ability of polymyxin B to interact with lipopolysaccharides and disrupt the membrane more effectively [27], while cold‐induced changes in outer‐membrane permeability have been shown to increase susceptibility to glycopeptides such as vancomycin [28]. In addition, thermal shifts rapidly alter DNA supercoiling and transcriptional activity processes controlled by DNA topoisomerases and RNA polymerase, the primary targets of fluoroquinolones and rifampicin, respectively [17, 29, 30]. Together, these observations suggest that temperature may also influence how transcription‐targeting antibiotics act within the cell. We therefore hypothesize that thermal shifts modulate gene responses to transcription‐targeting antibiotics by altering the physiological context in which these drugs exert their effects.

**FIGURE 1:**
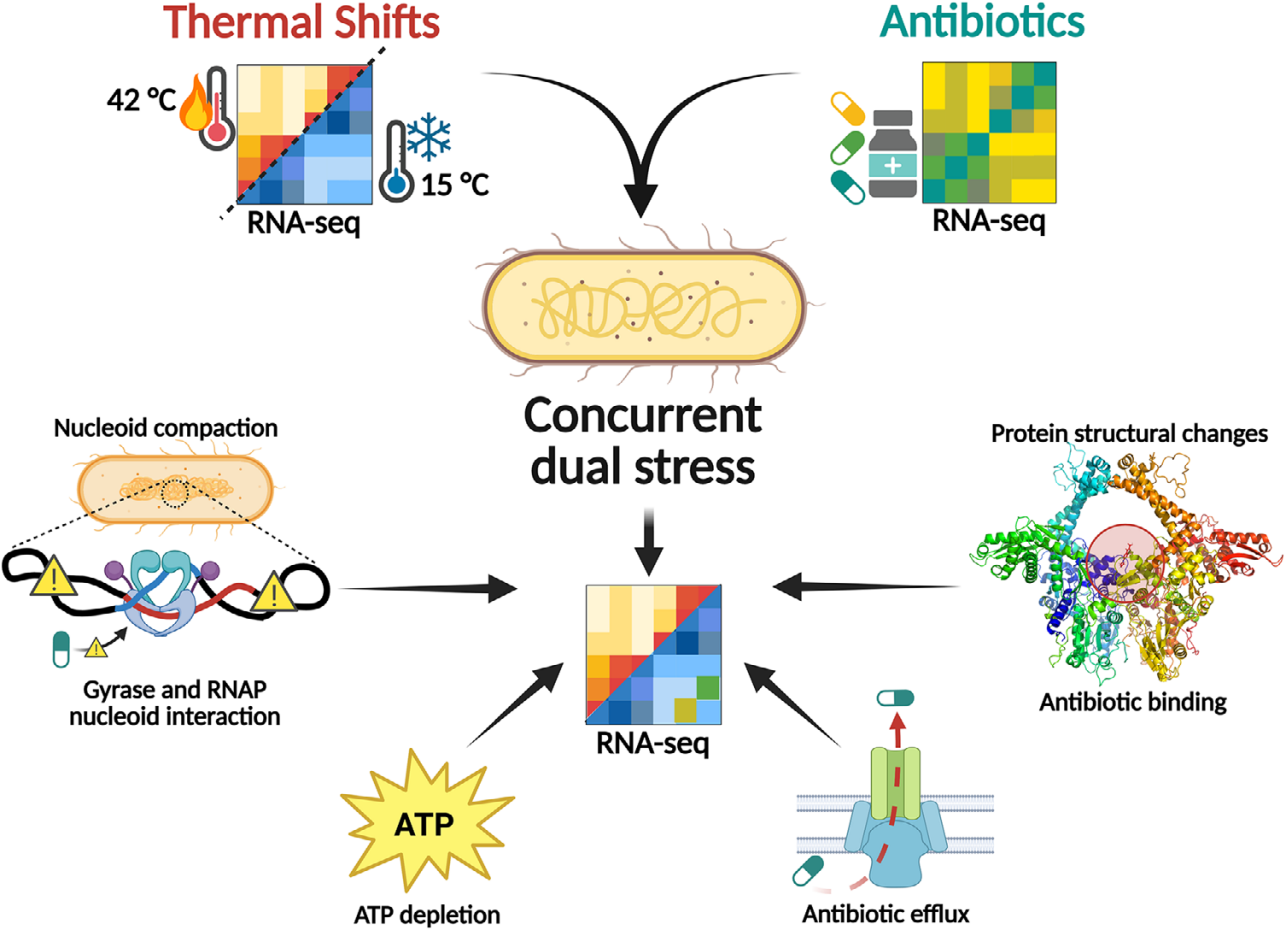
Conceptual overview of dual stress integration in *E. coli*. Thermal and antibiotic perturbations were applied individually or concurrently to examine the underlying response mechanisms. The schematic illustrates how multiple physiological layers converge to shape the bacterial response to dual stresses.

Here, we test this hypothesis and investigate the underlying mechanisms at the single‐gene level. We combine genome‐wide transcriptomics, metabolic profiling, nucleoid imaging, efflux induction assays, and molecular dynamics simulations to compare dual exposures with individual perturbations. We first examined canonical cold‐ and heat‐shock conditions and then expanded the analysis across intermediate temperatures and increasing antibiotic concentrations to define how the combined response varies across the parameter space. We further assessed whether these responses extend beyond *E. coli* by comparing functionally orthologous temperature‐responsive genes across evolutionarily distant bacterial species. Together, our results show that temperature shifts impose a fundamental constraint on antibiotic susceptibility in *E. coli* (Figure 1), particularly outside the intermediate temperature range, and that this response is partially conserved across diverse bacterial species.

## 2 Results

### 2.1 Global Transcriptional Responses to Individual Antibiotic and Temperature Stresses

**FIGURE 2:**
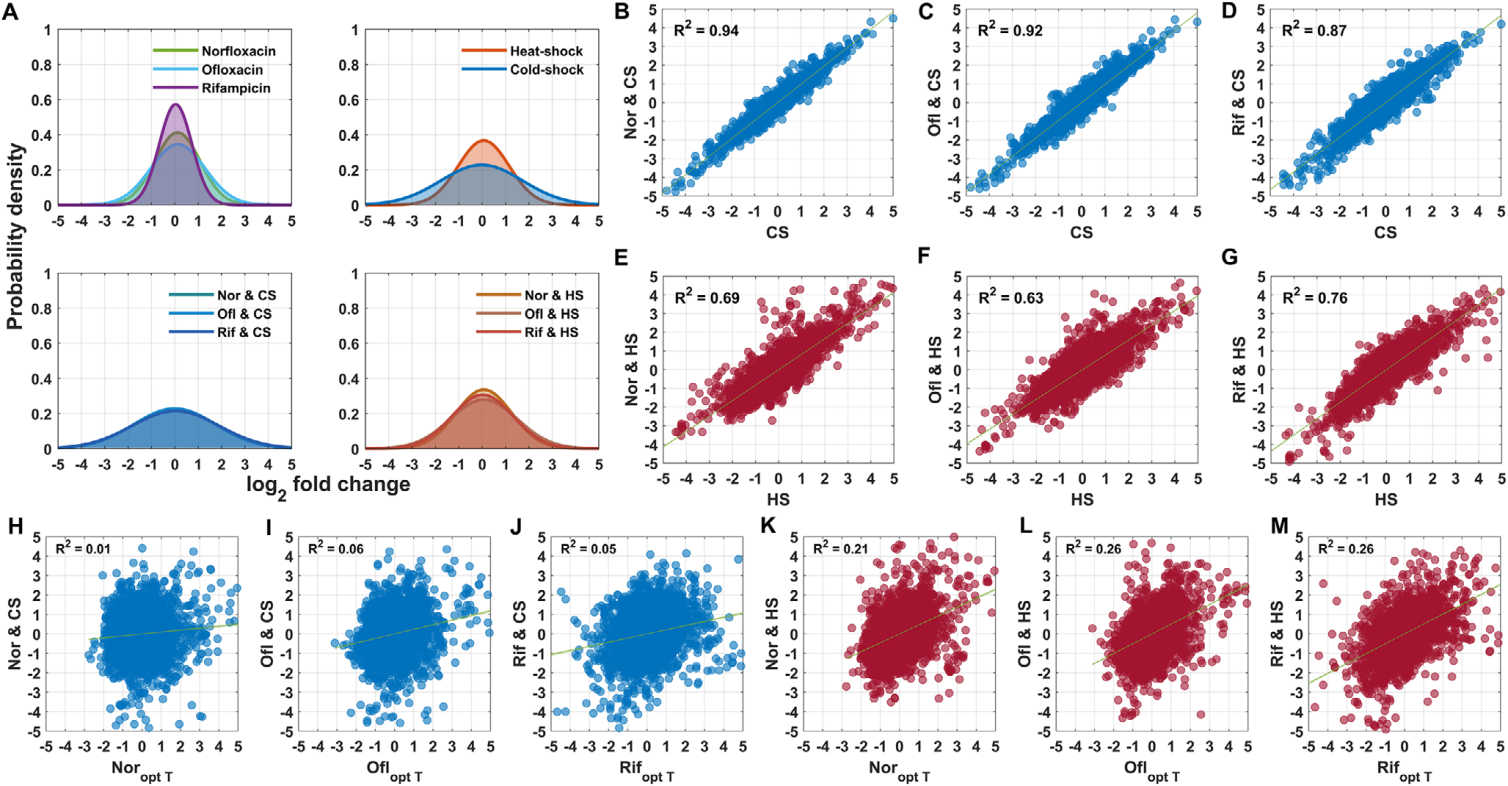
Temperature effects dominate the global transcriptional responses to dual stress. (A) Probability density distributions of log_2_ fold‐changes (LFC) under antibiotics (norfloxacin (Nor), ofloxacin (Ofl), rifampicin (Rif)), temperature shifts (heat (HS) or cold shock (CS)), and combined stresses. (B–G) Pairwise correlations between dual‐stress and temperature‐only profiles. Each point represents a gene; the green line shows the best‐fit linear regression with shaded 95% confidence intervals. (H–M) Pairwise correlations between dual‐stress and antibiotic‐only profiles. Each point represents a gene; the green line shows the best‐fit linear regression with shaded 95% confidence intervals. Also shown is the coefficient of determination (R^2^) for statistically significant correlations (*p* < 0.05). LFC values were z‐score normalized prior to the analysis. All inclinations are statistically significant (*p* < 0.05).

We began by targeting two essential, temperature‐sensitive processes, DNA topology and transcription [4, 5, 6, 7, 8], using fluoroquinolone and rifamycin antibiotic classes (Methods 5.1). Norfloxacin and ofloxacin, both fluoroquinolones, inhibit DNA gyrase and topoisomerase IV [16, 17], ATP‐dependent enzymes that regulate DNA supercoiling during replication and transcription by stabilizing cleavage complexes and generating DNA breaks [31, 32]. In contrast, rifampicin, which is generally considered bacteriostatic in *E. coli*, targets the β‐subunit (RpoB) of RNA Polymerase (RNAP), thereby preventing promoter clearance and transcription initiation [15]. Transcriptomic profiling (Methods 5.2) showed that norfloxacin and ofloxacin induced broadly overlapping, but not identical, responses (Figure 2A and Figures S1A and S2A), consistent with their similar mode of action. Meanwhile, rifampicin triggered a markedly different response (Figure 2A and Figures S1A and S2B,C).

We next examined the effects of thermal perturbations in *E. coli*. Both cold and heat shock triggered widely different responses (Figure 2A and Figures S1B and S3), as expected, given their fundamentally different physiological challenges [2, 3, 4, 5, 6]. Finally, direct comparison of antibiotic‐ and temperature‐ induced responses further showed that no antibiotic profile resembled either cold‐ or heat‐shock responses (Figures S1C,D and S4), indicating that antibiotics and thermal shifts activate largely non‐overlapping regulatory programs.

### 2.2 Temperature Effect Dominates Transcriptomic Responses Under Concurrent Dual Stresses

Having observed divergent responses to antibiotic and thermal stresses individually, we next examined how *E. coli* responds to concurrent (“dual”) temperature and antibiotic stresses (Methods 5.2). Strikingly, rather than producing a unique response, the dual‐stress transcriptomes closely resembled their corresponding temperature profiles (Figure 2A). Nevertheless, a few genes remained responsive to antibiotic cues, within the temperature‐dominant state. Scatter plots confirmed this trend (Figure 2B–M), and principal component analysis (PCA) (Methods 5.3) further supported this observation, with the first principal component capturing most of the variance (Figure S5), indicating that dual‐stress samples tightly clustered with their corresponding temperature‐only conditions. Consequently, the antibiotics that showed little correlation with each other (e.g., norfloxacin vs. rifampicin) became substantially correlated once combined with temperature stresses (Figure S6). Together, these findings show that, under the tested cold‐ and heat‐shock conditions, the combined transcriptional responses more closely resembled the corresponding temperature‐only responses than the antibiotic‐only responses.

### 2.3 Temperature‐Dominant Genes are Associated With Metabolic Regulation

We next investigated whether the same genes consistently exhibited temperature‐like responses across different antibiotic conditions. Genes whose dual‐stress expression remained more similar to the corresponding temperature‐only condition than to the antibiotic‐only condition across the tested antibiotics were defined as temperature‐responsive ‘shifters’.

**FIGURE 3:**
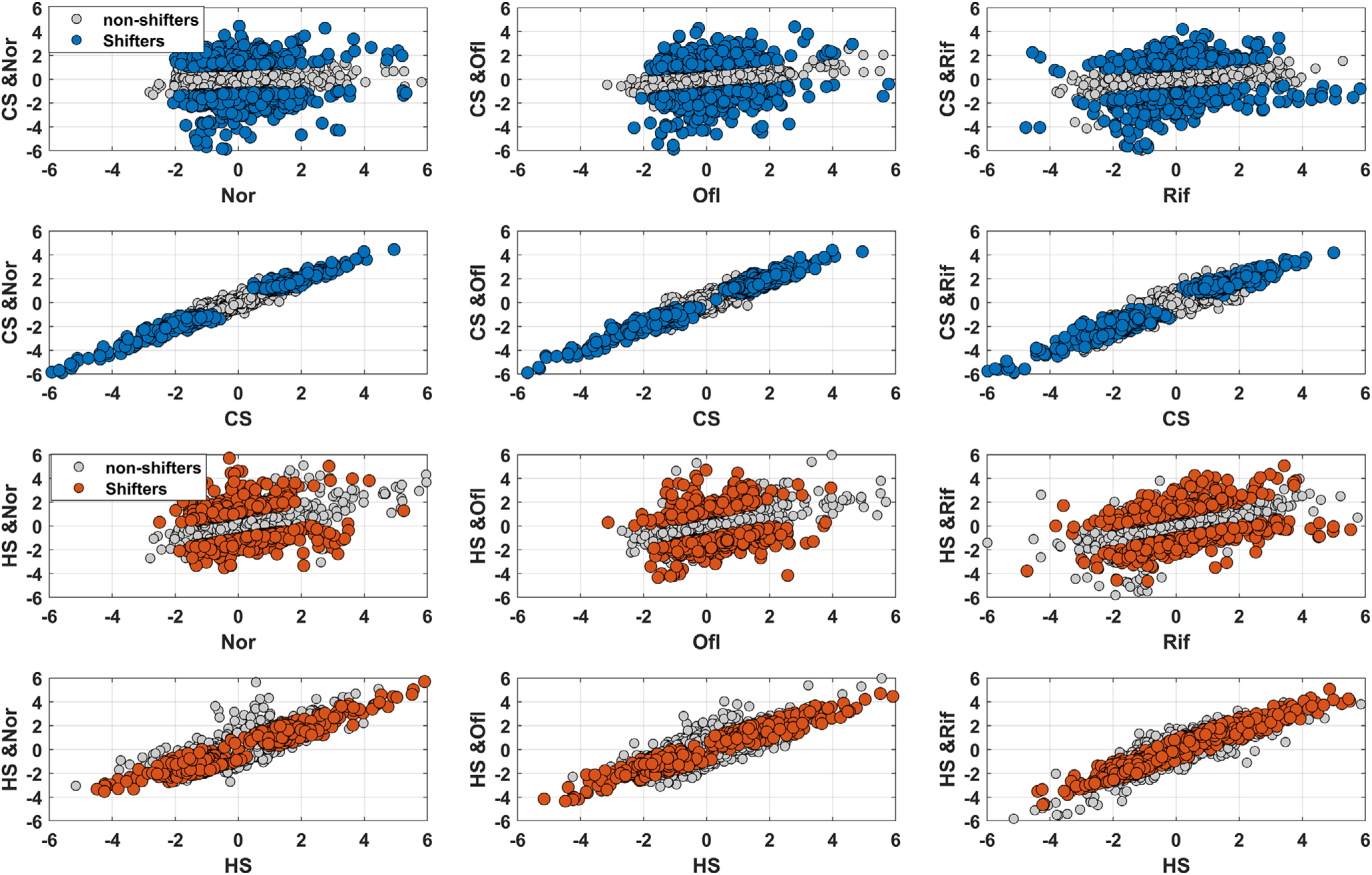
Shifter genes during dual stress. Scatter plots of log_2_ fold change (LFC) values under dual stresses vs. antibiotics or thermal shifts. Shifter genes are defined as those whose dual‐stress responses diverge from antibiotic responses but converge on thermal‐shift responses (Methods 5.4). Shifter genes are highlighted in blue (cold shock) and red (heat shock), while non‐shifters are shown in grey. Each point represents a gene.

Our regression‐based framework (Methods 5.4) identified 523 cold‐shock‐specific shifters and 286 heat‐shock‐specific shifters (Figure 3 and Figure S7). KEGG pathway enrichment analysis (Methods 5.5) of these genes revealed significant enrichment of distinct metabolic pathways (Figure S8). Cold shock‐specific shifters were enriched in oxidative phosphorylation and other central metabolic pathways, whereas heat‐shock‐specific shifters were primarily enriched in carbon metabolism and redox‐associated pathways. Thus, although cold and heat shock affected distinct metabolic pathways, both shifter gene cohorts were associated with processes capable of altering cellular energy homeostasis.

### 2.4 Cellular ATP Levels Decrease Under Thermal and Dual Stress But Not Under Antibiotic Stress

To contextualize these pathway‐level associations with the transcriptional responses, we performed Gene Ontology enrichment on the strongly downregulated and upregulated gene clusters (Methods 5.6–5.8). Strongly upregulated genes showed non‐overlapping enrichments in alternative carbon and nutrient utilization pathways, reflecting temperature‐specific adaptations (Figures S9–14). In contrast, strongly downregulated genes were enriched in aerobic respiration, oxidative phosphorylation, and other central energy‐generating pathways under both thermal‐stress conditions.

**FIGURE 4:**
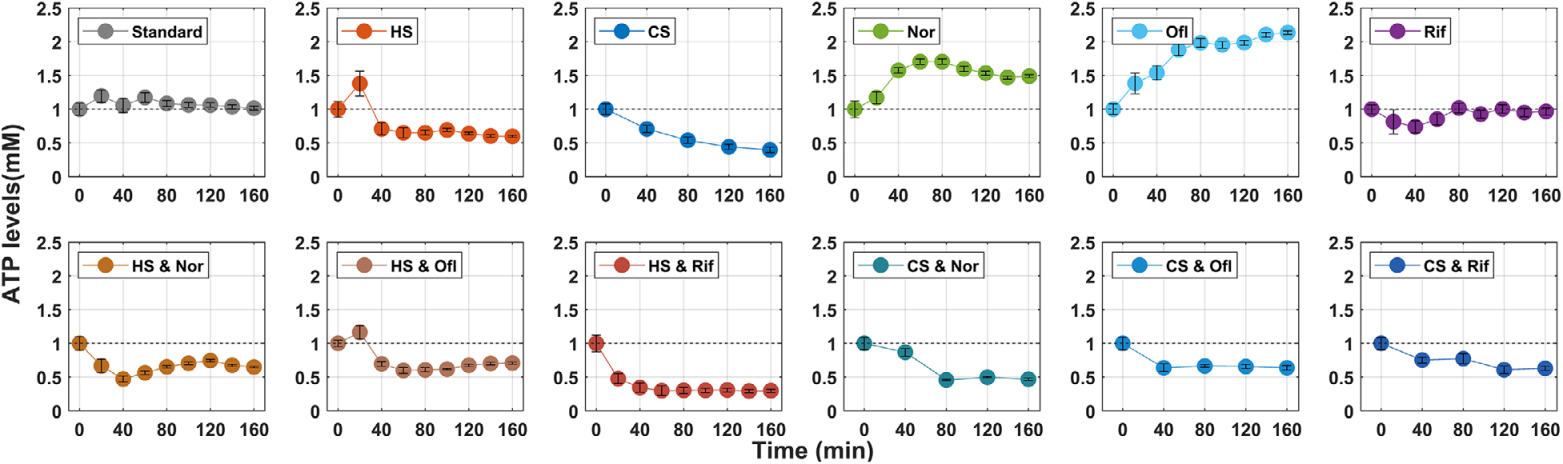
Thermal shifts influence ATP levels during dual stress. ATP concentrations measured over time using QUEEN‐2m cells [36] following antibiotic, temperature, and dual‐stress perturbations. Error bars (small) represent the standard error of the mean (SEM) from three biological replicates. The grey dotted line indicates the baseline (no change). For cold shock, measurements were taken every 40 min due to technical constraints. Values are normalized to time zero in each condition.

Because these processes directly contribute to ATP generation and cellular energy balance, we tested whether their transcriptional alteration was accompanied by changes in intracellular ATP levels (Methods 5.9). From Figure 4, ATP levels were stable under optimal conditions but were reduced under cold‐ and heat‐shock. In heat shock conditions, there was only a transient early increase, as previously observed [6, 33]. In contrast, fluoroquinolones increased ATP, whereas rifampicin remained largely neutral aside from a small transient decrease, in agreement with [18, 34]. This increase is in line with the previously reported increase in metabolic activity in cells subject to fluoroquinolones [19].

Interestingly, under all dual stresses, ATP levels decreased as if only the temperature shifts occurred (Figure 4), consistent with the transcriptomic and functional enrichment analysis. Given the above, and since the efficacy of fluoroquinolones scales with cellular metabolic activity [19, 29, 30, 35], this ATP reduction is in line with the temperature‐specific responses dominating during the dual stresses. Determining if this reduction under the dual stresses is due to disrupted metabolic pathways, impaired oxidative phosphorylation, and/or accelerated energy consumption (among other mechanisms) will require future studies.

### 2.5 Temperature Stress Impairs Nucleoid Association of Antibiotic Targets

Because transcription consumes (directly and indirectly) a substantial fraction of cellular ATP [37, 38], and its homeostasis relies on the interplay between supercoiling regulators and transcriptional machinery [6, 20, 39], we hypothesized that temperature‐induced metabolic downshifts could have downstream effects on the engagement of these regulators with the DNA.

**FIGURE 5:**
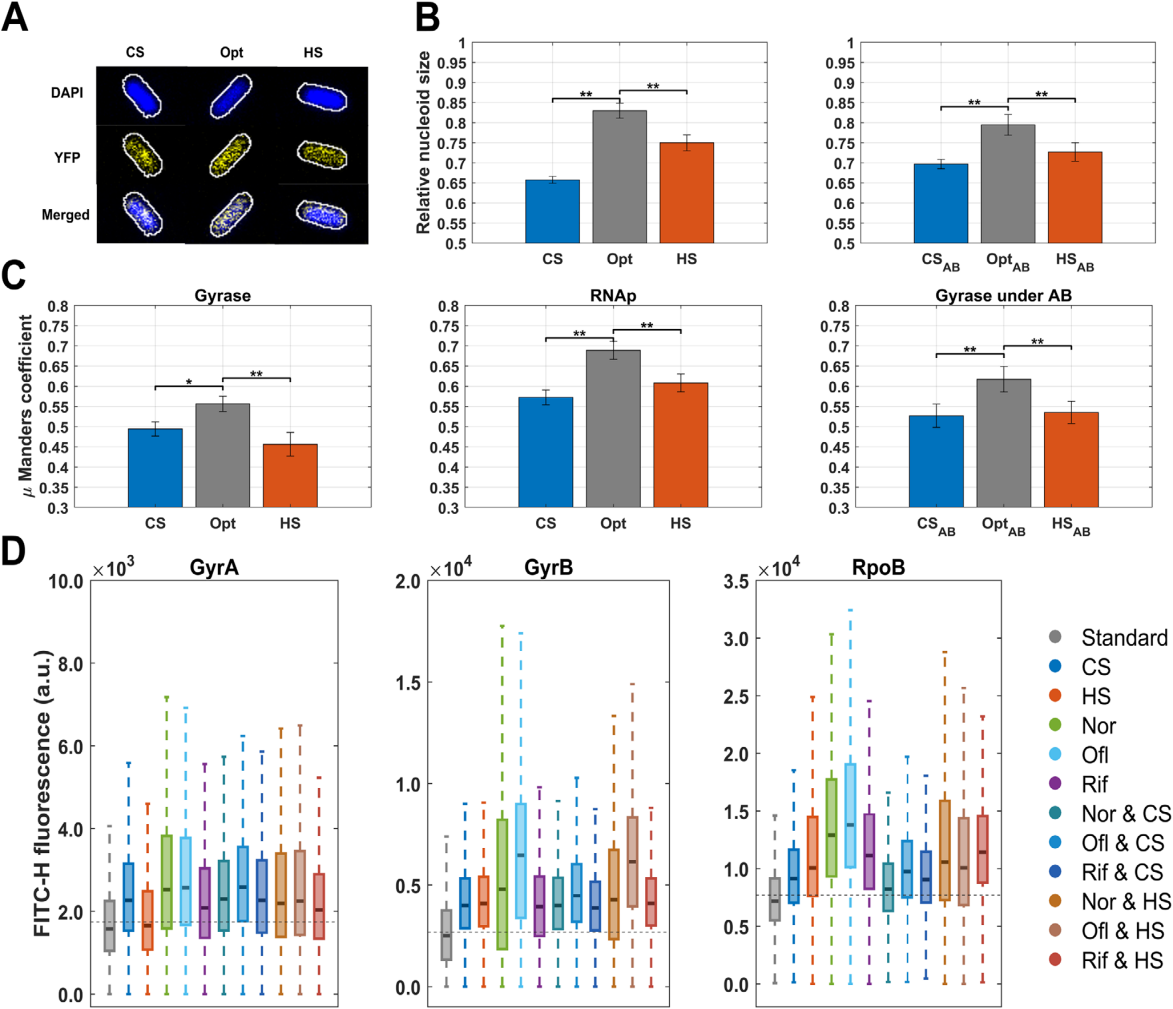
Thermal stresses alter the nucleoid colocalization and abundance of Gyrase and RNAP. (A) Example microscopy images of cells expressing GyrA‐YFP. Shown are 4′,6‐diamidino‐2‐phenylindole (DAPI)‐stained nucleoids (blue; top), GyrA‐YFP fluorescence (yellow; middle), and the merged DAPI/GyrA‐YFP images (bottom). White outlines indicate the cell boundaries derived from phase‐contrast images. (B)  Relative nucleoid area normalized by cell area as measured by fluorescence confocal microscopy, under cold shock, heat shock, and optimal conditions (left), and under their corresponding ofloxacin‐treated conditions (right). Error bars denote the SEM. (C) Colocalization of Gyrase and RNAP with the nucleoid, as quantified by µ (Manders’ coefficient) under cold‐shock, optimal, and heat‐shock conditions. The right panel shows Gyrasenucleoid colocalization under the corresponding ofloxacin‐treated conditions. Error bars denote SEM. (D) Single‐cell protein abundance of gyrase and RNAP measured by flow cytometry under antibiotic, temperature, and dual‐stress perturbations. The black dotted line indicates the mean fluorescence under standard conditions for each protein. In each box, the central line indicates the median, while the lower and upper edges correspond to the 25th and 75th percentiles, respectively. Statistical significance was assessed using a two‐tailed Welch's *t*‐test. *p* < 0.05 (^*^) and *p* < 0.01 ^(**^).

To test this, we performed microscopy (Methods 5.10) of fluorescently tagged DNA gyrases (gyrA) and RNAP's (RpoB), the primary targets of fluoroquinolones and rifampicin, respectively (Figure 5A and Figure S15). First, we observed a reduction in nucleoid size under both thermal and dual stress conditions (Figure 5B). Next, both GyrA and RpoB showed a reduction in nucleoid colocalization under both thermal shifts from (Figure 5C), suggesting altered spatial association of these proteins with the nucleoid (Methods 5.11). GyrA also showed reduced nucleoid colocalization under ofloxacin‐treated thermal stress conditions (Figure 5C), indicating that this altered spatial association persisted during antibiotic exposure. This may be a consequence of several phenomena, including temperature‐induced changes in nucleoid size and architecture, along with reduced enzymatic activity due to ATP depletion. Since the antibiotics' efficacy depends on the DNA‐bound conformation of these targets [15, 16], a thermal stress‐induced disengagement provides a plausible biophysical explanation for the reduced drug effects under dual stresses.

Given the spatial heterogeneity in RNA polymerase occupation of the nucleoid reported in [40], we also performed a different, indirect estimate of the effects of temperature shifts on the DNA engagement by RNAPs. Specifically, using τ plots, for each temperature, we estimated the effective RNAP‐promoter engagement time at a synthetic chromosomal integrated promoter during transcription initiation. The method was first proposed in [41] and later adapted to in vivo techniques [42]. For a detailed description, see (Methods 5.1). As shown in Figure S16, the estimated RNAP‐promoter engagement time was lower under both temperature‐shift conditions than under the standard condition. These results independently support the microscopy observations that temperature shifts alter RNAP association time with the DNA.

### 2.6 Gyrase and RNA Polymerase Levels Increase Under All Stress Conditions

In addition to interactability, cells may influence antibiotic efficacy by modulating the intracellular abundance of their targets. Prior studies have reported an inverse relationship between drug efficacy and target amplification; the rationale is that higher target concentrations require proportionally higher drug levels for effective inhibition [43]. We therefore quantified the single‐cell protein levels of DNA Gyrase (GyrA, GyrB) and RNAP (RpoB) (Methods 5.12).

Figure 5D shows that both targets are moderately induced across all stress conditions relative to their respective controls. This target induction supports the amplification model and likely contributes to the reduced antibiotic efficacy during dual stress. Notably, we also observed a linear correlation between the fluorescent signals obtained from microscopy and flow cytometry for each of these proteins (Pearson's r = 0.81, *p* < 0.05). However, average protein abundance did not correlate with nucleoid engagement (r = 0.21, *p* > 0.1), suggesting that protein production and engagement are uncoupled.

### 2.7 Thermal Stresses Alone Activate Antibiotic Resistance Regulators and Efflux Pump Genes

**FIGURE 6:**
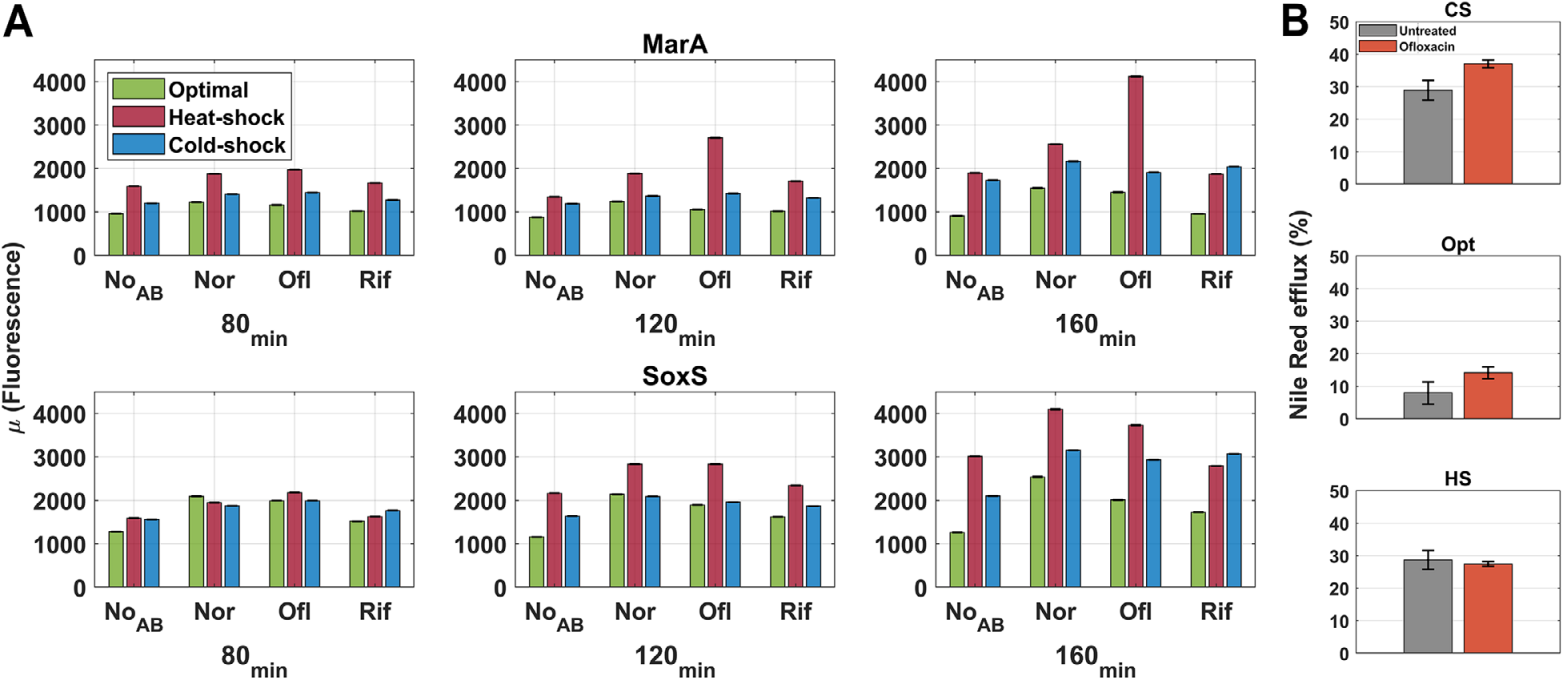
Temperature stress amplifies the resistance regulators' expression levels. Mean fluorescence levels of marA (top row) and soxS (bottom row) reporter strains from [48] measured by flow cytometry at 80, 120, and 160 min under standard, heat‐shock, and cold‐shock conditions, with or without antibiotics (norfloxacin, ofloxacin, rifampicin). Error bars denote SEM from three biological replicates. (B) Nile Red efflux activity measured under cold‐shock (CS), optimal (Opt), and heat‐shock (HS) conditions in untreated and ofloxacin‐treated cells. Efflux activity is expressed as the percentage of Nile Red efflux relative to the corresponding CCCP‐inhibited condition. Error bars denote SEM from biological replicates.

*E. coli* triggers complex regulatory responses to antibiotic exposure, and one of the best‐characterized is activation of the transcriptional regulators MarA and SoxS, which drive the AcrAB‐TolC efflux system [44, 45]. Consistent with previous reports [46, 47], our strain [48] showed moderate induction of both regulators under antibiotic stress at standard temperature (Figure 6A).

To determine whether thermal stresses alone influence this response, we measured marA and soxS expression under cold‐ and heat‐shock. At 160 min, heat shock increased marA and soxS transcript levels by approximately two‐ and threefold, respectively, while cold shock caused both genes to approximately double, when compared to standard conditions (Figure 6A).

Combining antibiotics with thermal stress further amplified these responses, with consistently higher expression levels over time and peaks at 160 min. On average, marA and soxS increased by ∼3.5 and ∼3‐fold under heat shock, respectively, and by ∼2 and ∼2.5‐fold under cold shock, relative to their antibiotic‐only conditions (Figure 6). RNA‐seq data of the efflux pump genes, acrA and acrB, confirmed this observation, showing significant upregulation (*p* < 0.05) under dual stresses compared with antibiotic‐only conditions (Figure S17).

To determine whether this transcriptional induction was accompanied by increased functional efflux, we directly quantified Nile Red efflux (Methods 5.12). In the absence of antibiotic treatment, both cold and heat shock showed higher Nile Red efflux than the standard 37°C condition (Figure 6B), indicating that thermal stress alone was associated with an elevated basal efflux state. Ofloxacin further increased efflux under cold shock and, to a lesser extent, at 37°C, whereas no additional increase was observed under heat shock. Consistent with the transcriptional data, these results show that thermal stresses alter both the regulation and the functional activity of the efflux system, although the magnitude of functional efflux did not scale uniformly with antibiotic exposure.

### 2.8 Protein‐Ligand Bonds Lose Stability Under Dual Stresses

Enzyme‐substrate binding is structurally independent of metabolic regulations, but sensitive to environmental changes, particularly thermal shifts. Both cold and heat shocks can alter protein folding, flexibility, and conformational equilibria [2, 4, 5, 49], potentially impacting antibiotictarget binding and efficacy.

To investigate this, we docked (Methods 5.13) norfloxacin, a representative fluoroquinolone, to its primary targets, DNA gyrase (PDB: 6rku) and topoisomerase IV (PDB: 1zvu). The best docked positions within the canonical binding pockets of the GyrA and parC subunits yielded predicted binding energies of −7.32 and −7.85 kcal/mol, respectively. For rifampicin, which binds to the β‐subunit (RpoB) of RNAP [17], we used the available co‐crystal structure (PDB: 4kmu). Each complex was then subject to all‐atom molecular dynamics (MD) simulations in GROMACS v2019 [50] under cold, standard, and heat‐shock conditions (Methods 5.14).

**FIGURE 7:**
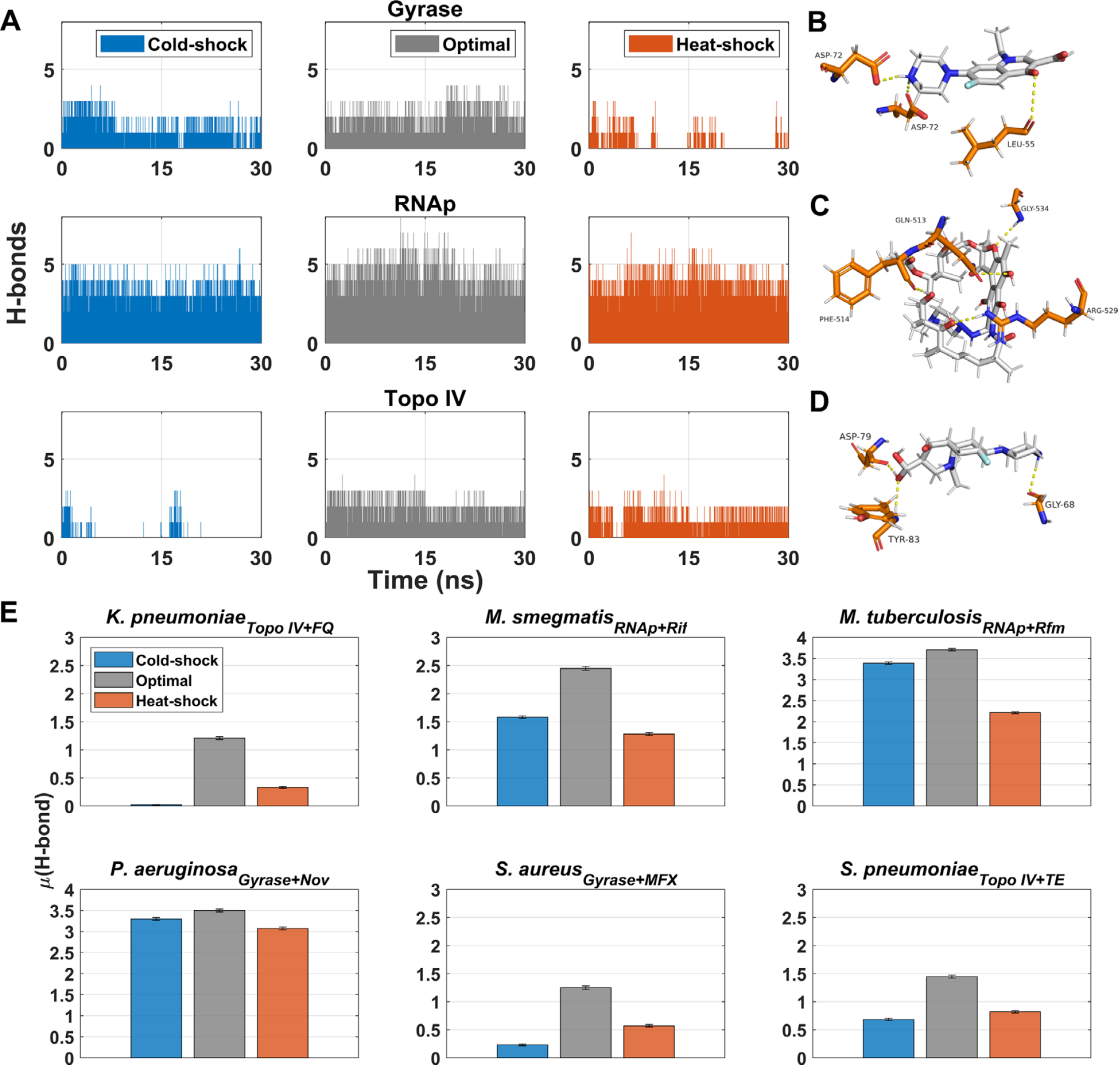
Thermal stresses alter the antibiotic‐target interactions during molecular dynamics simulations. (A) Time‐resolved hydrogen bond profiles between *E. coli*’s norfloxacin‐DNA gyrase, rifampicin‐RNAP, and norfloxacin‐topoisomerase IV. Results for cold shock, standard, and heat‐shock over 30 nanoseconds (ns). Each bar indicates the number of hydrogen bonds detected per frame. (B–D) Representative binding poses obtained as the centroid of the largest RMSD cluster under standard conditions for *E. coli*: (B) norfloxacin‐gyrase, (C) rifampicin‐RNAP, and (D) norfloxacin‐topoisomerase IV. Under cold (topoisomerase IV‐norfloxacin) and heat (gyrase‐norfloxacin) shocks, ligands dissociated from the binding pocket. Therefore, only standard conditions are shown. Hydrogen bonds are shown as dashed yellow lines. (E) Mean number of hydrogen bonds between antibiotics and their primary targets across multiple bacterial species (see labels) during 30‐ns MD simulation, under cold, standard, and heat‐shock conditions. Error bars represent the standard error of the mean (SEM).

Root Mean Square Deviation (RMSD) analysis of the backbone confirmed overall protein stability across the 30‐nanosecond trajectories, though with larger fluctuations at elevated temperatures (Figure S18). Inter‐molecular Hydrogen bond occupancies, a proxy for structural stabilization, were maximized under standard conditions (Figure 7A and Table S1), with stable interactions maintained at active‐site residues. Structural snapshots of the binding sites under standard conditions (Figure 7B–D) confirmed these trends. Heat shock disrupted key gyrase‐norfloxacin hydrogen bonds, consistent with increased conformational entropy [2, 51, 52]. Meanwhile, cold shock caused ligand dissociation from the topoisomerase IV binding cavity.

Finally, as an orthogonal metric of binding stability, we calculated short‐range nonbonded interaction energies using Coulombic and van der Waals components. Consistent with the H─bond analysis, the most favorable interaction energies were observed under standard conditions, with both cold and heat shock hampering drug‐target interactions (Figure S19 and Table S2). Together, these results demonstrate that thermal fluctuations impair the antibiotic‐enzyme interactions by reshaping the free‐energy landscape of the antibiotic‐target complexes, which destabilizes the hydrogen bonding networks and reduces the favorable nonbonded interactions.

### 2.9 Cross‐Species Conservation of Temperature‐Dependent Responses

DNA Gyrase and RNAP share highly conserved architectures and catalytic cores across bacterial phyla [53, 54]. Consistent with this, our phylogenetic analysis identified more than 1150 homologs of these targets (Methods 5.15). To test whether such conservation is also reflected biophysically, we searched the NCBI database for species with experimentally verified protein structures in complex with fluoroquinolones or rifamycin antibiotic classes. We selected representatives from both Gram‐positive and Gram‐negative bacteria with diverse pathogenic profiles. Notably, these co‐crystal structures involved ligands that differed from those in *E. coli*, providing distinct antibiotic‐target contexts. For each species, an ortholog (either Gyrase or RNAP) was simulated with its corresponding antibiotic under cold‐shock, standard, and heat‐shock conditions for 30 ns using GROMACS [50].

Across all species, standard temperature consistently favored the most stable hydrogen bond formation between ligand and target (Figure 7E and Table S3). However, the magnitude of disruption under stress conditions varied in a species‐specific manner, suggesting differences in their conformational plasticity (Figure S20). For instance, RNAP‐rifampicin complexes in *Mycobacterium smegmatis* and *Mycobacterium tuberculosis* remained relatively stable under cold and heat shock, with fewer hydrogen bonds than in the standard condition. However, Gyrase‐antibiotic complexes in *Streptococcus pneumoniae* and *Klebsiella pneumoniae* showed significant differences to thermal shifts. Interestingly, the *Pseudomonas aeruginosa* Gyrase‐novobiocin complex maintained moderate stability, even under thermal shifts. This is likely due to the distinct mode of action of novobiocin, which competitively inhibits ATP binding, as opposed to the fluoroquinolones, which primarily target the subunit *GyrA* [20]. Together, these findings suggest that, although species‐specific features modulate the magnitude of the disruption, the overall trend is conserved. Thus, the thermal modulation of antibiotic‐target interactions appear to be a broadly conserved feature, rather than an idiosyncratic property of *E. coli*.

Next, our phylogenetic analysis further revealed that 5200 bacterial species contain ≥ 5 conserved *E. coli* genes. Moreover, the shifter genes were conserved in an average of 448 species, which is more than twice the number expected from random gene cohorts of the same size [210]. However, the presence of homologous genes across species does not necessarily establish conservation of their temperature‐dependent transcriptional responses.

To test whether the shifter response extends beyond *E. coli*, we analysed publicly available transcriptomic datasets from a panel of pathogenic and environmental bacteria exposed to comparable cold‐ or heat‐shock conditions. Functional counterparts of the *E. coli* shifter genes were identified using KEGG Orthology assignments, allowing genes to be matched through their shared functional ortholog groups (Methods 5.15).

For each orthologous gene‐species pair, the direction of the temperature‐induced log_2_ fold change was compared with the corresponding *E. coli* shifter gene. Responses were classified as concordant when the ortholog changed in the same direction as in *E. coli* and discordant when in the opposite case. Genes without an identifiable functional ortholog or available expression data were treated as unavailable and excluded from the calculation.

**FIGURE 8:**
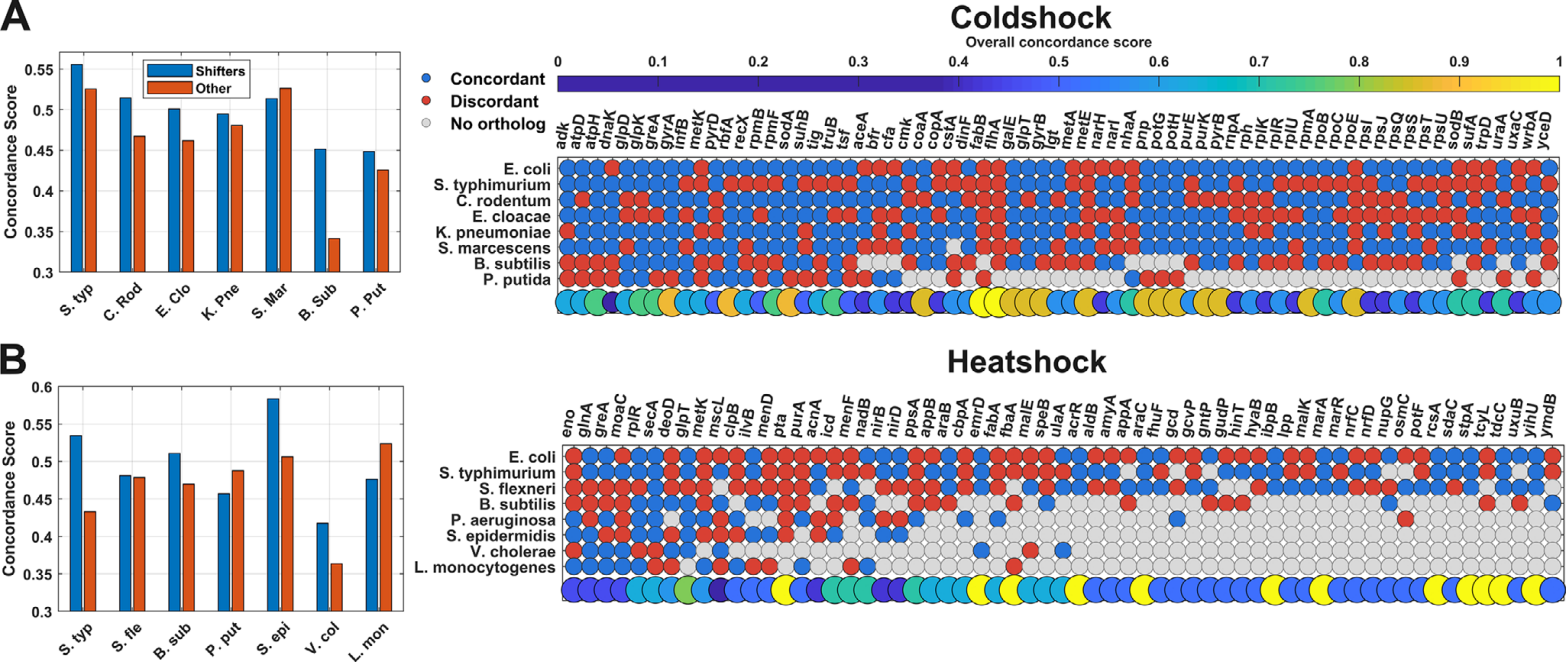
Cross‐species expression concordance of shifter genes. (A, B) Bar plots show the mean concordance score of the shifter cohort and the remaining temperature‐responsive genes in each species under cold shock (A) and heat shock. (B) The ortholog‐level matrices show the direction of the expression response of individual shifter genes across species. Blue circles indicate responses concordant with the corresponding *E. coli* gene, red circles indicate discordant responses, and grey circles indicate the absence of an identifiable functional ortholog or an unavailable expression measurement. The bottom row shows the overall gene‐level concordance score, calculated as the fraction of orthologs displaying the same response direction as the corresponding *E. coli* genes. Circle color represents the concordance score from 0 to 1.

For each species, we calculated a species‐level concordance score as the fraction of genes evaluable whose response direction matched the observed in *E. coli*. This score was calculated separately for the shifter cohort and for the remaining temperature‐responsive genes. Under cold shock, the shifter cohort showed a higher concordance than the temperature‐responsive genes in most species examined, including *Salmonella Typhimurium*, *Citrobacter rodentium*, *Enterobacter cloacae*, *Klebsiella pneumoniae*, *Bacillus subtilis*, and *Pseudomonas putida* (Figure 8A). The difference was smaller or reversed in some species, including *Serratia marcescens*, indicating that response conservation is not universal. A similar, but more variable pattern was observed under heat shock when compared to *Salmonella Typhimurium*, *Shigella flexneri*, *Bacillus subtilis*, *Pseudomonas aeruginosa*, *Staphylococcus epidermidis*, *Vibrio cholerae*, and *Listeria monocytogenes*. Across the species, shifter genes displayed equal or higher concordance than the non‐shifter background across the species, although the magnitude of the difference varied between organisms (Figure 8B). We additionally calculated a gene‐level concordance score for each *E. coli* shifter gene as the fraction of evaluable species in which the corresponding ortholog changed in the same direction. The resulting matrix showed that some shifter responses were retained across several species, whereas others were restricted to a narrower phylogenetic range or could not be evaluated because an ortholog or corresponding expression measurement was unavailable.

Together, these results suggest that a subset of functionally orthologous genes retains a concordant response across diverse pathogenic, environmental, Gram‐negative, and Gram‐positive bacteria. However, the incomplete concordance and species‐specific deviations suggest a partial, rather than universal, conservation.

### 2.10 Mapping Cellular Responses Across the Temperature‐Antibiotic Parameter Space

To determine whether the observed temperature‐associated dominance was specific to the selected temperatures and AB concentrations tested above, we expanded the conditions to also study intermediate temperatures and antibiotic concentrations. For this, we measured the expression levels of ten representative genes (selected from the shifter genes cohort) across six temperatures and three ofloxacin concentrations, with the highest concentration corresponding to approximately 20 × the minimum inhibitory concentration (MIC)(Figure S21). For each combined condition, the responses were classified as temperature‐ or antibiotic‐like by comparing their differences in the response from their corresponding temperature‐only and antibiotic‐only responses, respectively (Methods 5.16).

**FIGURE 9:**
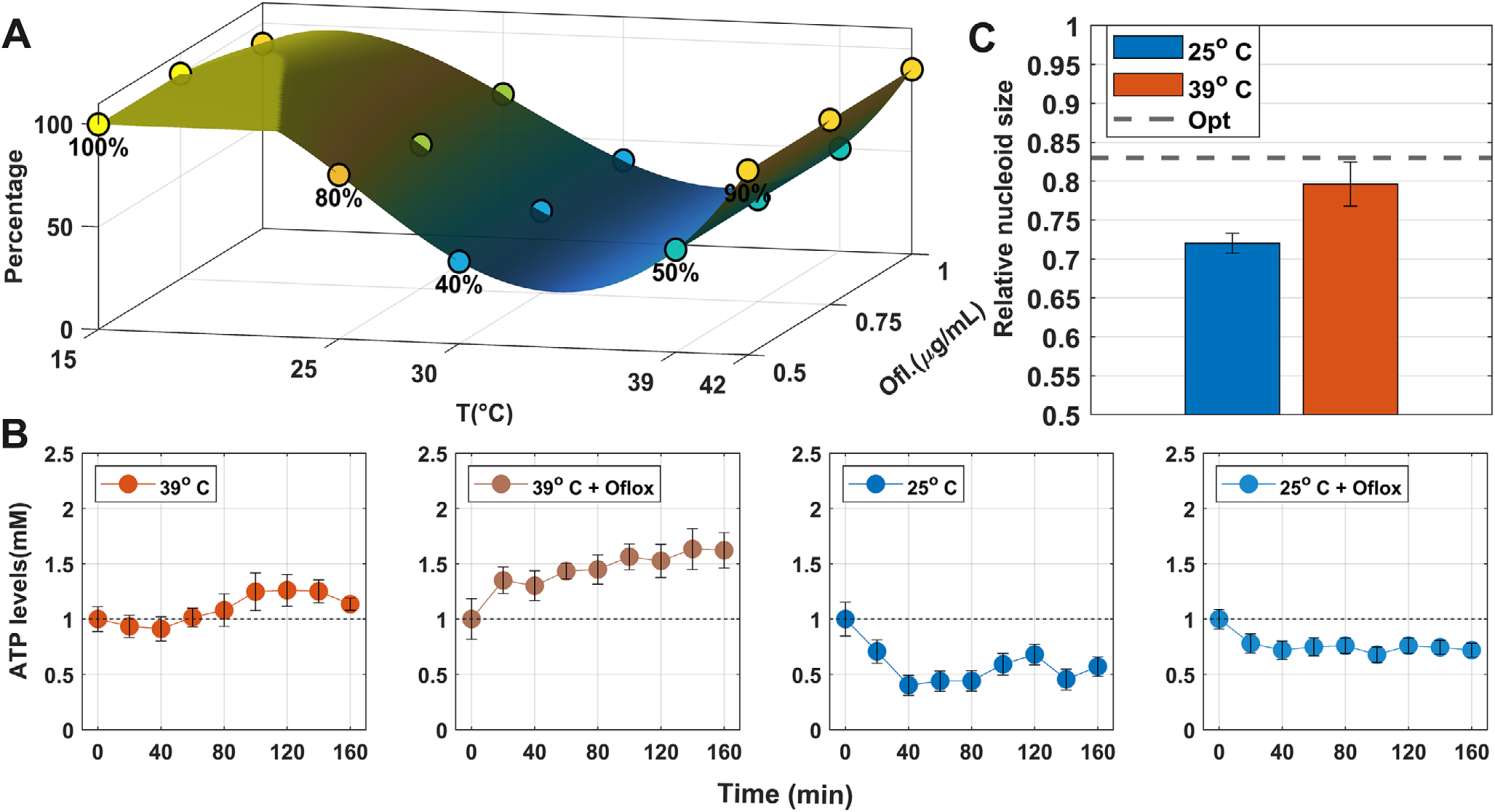
Temperature‐dependent cellular responses across intermediate thermal and ofloxacin conditions. (A) Percentage of reporter genes classified as temperature‐like across the tested temperature‐ofloxacin state space. Classification was based on the relative distance of each combined‐condition response from its corresponding temperature‐only and antibiotic‐only responses. The surface represents the overall temperature‐like fraction across ten representative shifter genes. (B) Intracellular ATP levels measured over time at 39°C and 25°C, in the absence or presence of ofloxacin. ATP levels were normalized to the corresponding initial value at 0 min. Error bars denote SEM. (C) Relative nucleoid size measured at 25°C and 39°C. The dashed line indicates the mean relative nucleoid size under the optimal 37°C condition. Error bars denote SEM. Statistical significance was assessed using a two‐tailed Welch's *t*‐test (unequal variances). Both 25°C and 39°C conditions were significantly different from the 37°C reference condition (*p* < 0.05).

The response curve shows that the temperature dominance depends primarily on the magnitude of the thermal shift from the standard growth condition. At temperatures below 30 or above 39, most reporters showed responses closer to their corresponding temperature‐only profiles. In contrast, within the intermediate range of 30°C–39°C, only approximately 50% of the reporters retained a temperature‐like response, indicating that their responses became gradually temperature‐like, rather than undergoing a rapid shift at a given temperature (Figure 9A and Figure S22). Interestingly, increasing the concentration of ofloxacin had little to no effect on this pattern, including the highest concentration tested, which was approximately 20x MIC.

Next, we investigated intracellular ATP levels at the intermediate temperatures. ATP levels at 25°C decreased toward those observed during 15°C cold shock, whereas ATP levels at 39°C remained comparatively close to the standard 37°C condition (Figure 9B). We then examined if the nucleoid architecture followed a similar pattern. Nucleoid size at 25°C was similar to that observed under cold‐shock conditions, whereas at 39°C it remained close to the standard 37°C condition (Figure 9C).

Finally, we also extended the temperature range of the molecular dynamics simulations for the gyrase‐norfloxacin and topoisomerase IV‐norfloxacin complexes. The gyrase‐norfloxacin complex remained comparatively stable within the lower and near‐optimal temperature regimes (15°C, 25°C, 34°C, and 37°C) but showed increasing destabilization beyond 39°C, as indicated by changes in hydrogen‐bond occupancy (Figure S23). Contrarily, the topoisomerase IV‐norfloxacin complex was less stable at low temperatures.

Overall, the effects of temperature were not uniform. Temperature‐associated changes in transcription rates, ATP levels, and nucleoid architecture became more prevalent outside the 30°C–39°C range. At the molecular level, temperature‐dependent changes in antibiotic‐target interactions were target specific, occurring preferentially at higher temperatures for gyrase and lower temperatures for topoisomerase IV.

### 2.11 Common Features of the Differentially Expressed Genes Under Single and Dual Stresses

Next, we investigated which potential regulatory features of *E. coli* most influence the convergence of dual‐stress transcriptomes with their respective thermal‐stress profiles. For this, we trained a Random Forest (RF) classifier machine learning model (Methods 5.17), using 34 features from RegulonDB [55], including gene essentiality, sigma factor preferences, supercoiling sensitivity, stringent response, and global transcriptional regulators. The model achieved prediction accuracies of ∼78% and ∼73% for cold and heat shock, respectively (Figure. S24).

Among others, σ factors, global transcription factor (TF) regulators, and nucleoid‐associated processes consistently ranked in the top 5 most informative predictors across both thermal stresses (Table 1). The relatively lower effect sizes (maximum importance of ∼4% in cold and ∼3.4% in heat shock, respectively), indicate that thermal dominance arose from a combination of regulatory mechanisms, rather than a single driver.

**TABLE 1:** Key regulatory features identified by random forest classification. Shown are the increase in out‐of‐bag (OOB) error rates when the feature is permuted (importance score), the probability of gene upregulation when the feature is present (P(upregulation|X = 1)), the probability of upregulation when the feature is absent (P(upregulation|X = 0)), and the difference between the two (Δ). A positive Δ indicates that the feature increases the likelihood of gene upregulation, whereas a negative Δ indicates reduced likelihood.

Feature	Increase in % of OOB error rates	P(upregulation∣X=1)	P(upregulation∣X=0)	Δ
Cold shock				
σ^70^	3.87	0.56	0.49	0.07
σ^38^	3.44	0.52	0.30	0.22
*CRP* (+)	3.31	0.50	0.29	0.21
*narL* (‐)	3.11	0.01	0.52	−0.51
Supercoiling sensitivity (SS)	2.67	0.35	0.51	−0.16
Heat shock				
*CRP* (+)	3.36	0.83	0.46	0.36
σ^38^	3.19	0.54	0.31	0.23
σ^70^	3.10	0.59	0.46	0.13
σ^32^	2.63	0.53	0.23	0.30
*ihfA* (+)	2.29	0.57	0.51	0.06

Notably, the stress‐shared contributors, such as σ^70^, σ^38,^ and CRP+, highlight pathways that buffer metabolic burden [35, 55, 56, 57], while context‐specific signals included supercoiling sensitivity under cold shock [6] and σ^32^ [1, 2, 4] under heat shock. Together, these findings reinforce our conclusion that temperature dominance emerges from the interplay between the mechanisms involved in metabolic control, DNA topology maintenance, and canonical thermal shift responses.

## 3 Discussion

By profiling *E. coli* transcriptomes following dual thermal and antibiotic perturbations, we have demonstrated that temperature is the dominant axis of stress response, particularly outside the intermediate 30°C–39°C range. This dominance emerges from a cascade of interconnected mechanisms.

First, dual stress was associated with a reduced energetic state, a phenomenon reported under thermal shifts (6, 33). Among others, thermal adaptations constrain energy availability through reduced growth, diminished flux, and altered membrane dynamics [5, 6, 8, 33]. In our case, growth‐rate effects can largely be excluded from this list, as the perturbations applied here were chosen to minimally influence growth, except under cold shock, which inherently slows proliferation below 30°C [6]. Because fluoroquinolones and rifampicin exert their action primarily on replication and transcription, which scales with metabolic output [15, 19, 29, 30, 34, 35], these temperature‐associated changes in cellular energy state may reduce transcriptional and replicative activity and thereby contribute to altered antibiotic responses.

Next, thermal shifts also remodeled the nucleoid architecture and altered the spatial association of DNA‐binding proteins with the nucleoid. Consistent with earlier reports [6], we observed modest nucleoid compaction together with reduced nucleoid colocalization of RNA polymerase and DNA gyrase. These changes may influence transcription‐associated supercoiling and the spatial organization of topoisomerase activity [39]. However, nucleoid colocalization does not directly measure DNA binding or target engagement; therefore, the extent to which these changes modify antibiotic action remains to be established.

At the molecular level, thermal shifts also reshaped the conformational plasticity of the targets, which can be explained by simple entropic forces. Cold likely restricts the degrees of freedom, reducing conformational flexibility [50], whereas heat increases entropic fluctuations [4, 49, 50]. Consistent with this, our simulations revealed temperature‐dependent changes in antibiotic‐target interactions, although the magnitude and direction of these effects varied among protein‐ligand complexes. These findings indicate that temperature can alter antibiotic‐target interaction stability in a complex‐specific manner.

At a systems level, *E. coli* exhibited another layer of defense against antibiotics. Our flow cytometry data showed that thermal shifts alone sufficed to amplify the expression of efflux regulators, such as *marA* and *soxS*, which are classically induced by antibiotics [46, 47]. Importantly, this transcriptional response was accompanied by increased functional Nile Red efflux. Notably, *marA* has been associated with membrane stabilization and response to pH stresses, while *soxS* is activated under oxidative stress [48, 58, 59, 60]. These associations suggest that the oxidative stress imposed during heat [4, 6], together with the altered membrane integrity and consequent pH stress under both cold and heat [3, 4, 5, 8, 61], may mediate this regulatory crosstalk, protecting bacterial cells from antibiotics during the dual stresses.

In addition, our machine‐learning analysis highlighted *CRP* as a key predictor of differential gene expression, pointing to elevated cAMP levels [56, 57] in the cell. This is consistent with the hypometabolic state that we observed, as CRP is central to activating alternative pathways during low metabolic flux [57], with *narL* and *ihfA* acting as *auxiliary* nodes in metabolic recovery in cold and heat conditions, respectively [62]. The predicted importance of σ^38^ further indicates that the cells might be shifting toward stationary‐phase‐like physiology, a state with reduced growth and antibiotic susceptibility [35, 56]. Overall, the modest and distributed contributions of the predictors suggest that the hypometabolic shift is not enforced by a single vulnerable node but instead arises from a layered regulatory program that collectively stabilizes temperature‐defined states.

Importantly, temperature‐responsive patterns were not unique to *E. coli*. Phylogenetic analysis first showed that “shifter genes” were broadly conserved across bacterial species. Cross‐species transcriptomic analysis further showed that functionally orthologous shifter genes exhibited higher mean concordance in the direction of temperature‐induced expression than other temperature‐responsive genes in several phylogenetically diverse bacterial species. However, the extent of concordance varied across species and individual genes, which may partly reflect differences in experimental design and growth conditions among the available datasets. Because this analysis examined the conservation of temperature responsiveness alone, it does not directly establish conservation of the shifter response under combined temperature‐antibiotic stress, which will require direct testing in future studies.

Finally, molecular‐dynamics simulations of Gyrase, Topoisomerase IV, and RNAP revealed temperature‐dependent changes across phylogenetically distant bacteria for diverse antibiotics, although the magnitude and direction of these effects varied among species and protein‐ligand complexes. Together, these findings support partial conservation of temperature‐responsive features across bacteria, while indicating that their expression and molecular consequences are species‐ and context‐dependent.

At the same time, the expanded temperature‐antibiotic parameter space further showed that temperature predominance was not uniform across all tested conditions. Instead, temperature‐like responses were more prevalent outside the intermediate 30°C–39°C range, whereas responses within this range were mixed, even as the ofloxacin concentration increased to approximately 20 × MIC. This pattern suggests that, once the thermal deviation is sufficient to engage cold‐ or heat‐associated adaptation programs, the resulting physiological state becomes increasingly shaped by temperature. Thus, the relative influence of temperature and antibiotic stress depended primarily on the magnitude of the thermal perturbations.

Although the study was limited to two classes of antibiotics, we expect that the findings apply to other antibiotics as well. In agreement, heat shocks have been shown to increase the frequency of persister and resistant cells [62, 63], and studies have revealed correlations between rising environmental temperature and antibiotic tolerance [64]. In this view, bacterial survival under antibiotics need not arise solely from genetic mutations but may also occur as a (potentially incidental) consequence of temperature‐dependent metabolic and biophysical reorganization.

Together, our results identify temperature as an important determinant of bacterial responses to transcription‐targeting antibiotics. However, the translational relevance of these findings to human infections remains to be further explored by future studies conducted within clinically relevant temperatures, therapeutic antibiotic exposures, and direct susceptibility measurements. We expect that these studies will show many genes being largely controlled by the temperature shift, providing a temporal window in which temperature‐induced physiological states facilitate adaptation to the antibiotic exposure and potentially increase the opportunity for resistance to emerge.

## 4 Conclusion

Our findings suggest that the effects of exposing bacteria to temperature shifts along with transcription‐targeting antibiotics are not additive. Instead, the resulting transcriptome largely resembles the one caused by thermal shifts alone. This response was associated with temperature‐dependent changes in cellular energy state, nucleoid organization, and the spatial association of DNA Gyrase and RNAP with the nucleoid, together with altered RNAP‐promoter engagement. Temperature also affected antibiotic‐target interactions and functional efflux activity, while several global transcriptional regulators were associated with the combined response. Importantly, the expanded parameter‐space analysis showed that temperature‐like responses were more prevalent outside the intermediate 30°C–39°C range and were more heterogeneous within this range. Overall, our findings highlight the importance of environmental factors in shaping bacterial adaptability to antibiotics.

## 5 Materials and Methods

### 5.1 Bacterial Strains, Growth, and Stress Conditions

We used *E. coli* K‐12 MG1655 as the wild‐type strain. From a glycerol stock (at −80°C), cells were streaked on LB agar plates and incubated at 37°C overnight. The next day, a single colony was picked from the plate, inoculated in fresh LB medium supplemented with appropriate antibiotics, and incubated at 37°C overnight with shaking at 250 RPM. Overnight culture cells were then diluted into fresh M9 medium and supplemented with 0.4% glucose, 1X Minimum Essential Medium (MEM) amino acids from Sigma–Aldrich, and 1X MEM vitamin solutions also from Sigma–Aldrich. Next, cells grew at 37°C with aeration, until reaching the mid‐exponential growth phase (OD_600_ of 0.3).

Stresses were then applied, and measurements were performed after cells were 160 min under stress. The following stresses were applied: (i) Cold shock was applied by placing cells at 15°C. At this temperature, cells no longer divide, albeit not shifted to stationary growth [6]. Lower temperatures were not applied, since it would increase harm to the cells. (ii) Heat shock was applied by placing cells at 42°C since higher temperatures damage protein structures [63].

For each AB, we applied the highest concentration at which cell growth was not significantly affected at the time the RNA‐seq and other measurements were performed (i.e., 160 min later or prior). However, these concentrations are nevertheless lethal if the cells are kept under stress for longer (e.g., 24 h), except for rifampicin, which is not bactericidal. This was tested by growing large batches of culture into mid‐exponential phase, dividing them into several tubes, and subjecting them to different antibiotic concentrations. In the end, the concentrations selected for this study were 0.10 µg/mL of norfloxacin, 0.5 µg/mL of ofloxacin, and 2.5 µg/mL of rifampicin, respectively.

Strains from a chromosomally integrated YFP fusion strain library from the *E. coli* Genetic Resources at Yale's Genetic Stock Center (https://ecgrc.net/CGSC/Strain.php?ID=134428) were used to track several genes.

To measure intracellular RNAP, we used an RL1314 strain with RpoC endogenously tagged with GFP (generously provided by Robert Landick). Also, we used an OSYM (WT) strain generously provided by the Elf lab with a chromosomally integrated, constitutive, synthetic promoter pFAB120 (35 bp long). It is followed by one LacI binding site (20–21 bp long), a double ribosome binding site, RBS (33 bp long), a mVenus coding sequence (717 bp), and a double terminator sequence (57 bp).

To obtain cells with different intracellular RNAP concentrations, we started from LB medium (1 g tryptone, 0.5 g yeast extract, and 1 g NaCl for 100 mL and pH 7.0). We produced this tailored medium following [42, 64] by reducing tryptone and yeast extract according to the fractions x0.8, x0.75, and x0.5, respectively, while keeping NaCl concentration unchanged. Both the RL1314 and the OSYM strains were placed in these media conditions to measure, respectively, the effects of different media on RNAP and on mVenus levels. In all cases, pFAB120 was fully induced by 1 mm of IPTG, added at 0.3 OD600. Measurements were performed at 0, 80, and 160 min after adding IPTG.

By combining the measurements of RNAP and fast‐maturing mVenus under the control of pFAB120, at different temperatures, we estimated the effects of temperature on the effective engagement of RNAP with the synthetic promoter using τ plots [41, 42]. Briefly, the mean expression rate between two consecutive time points was estimated as ((E_2_‐E_1_)/(t_2_‐t_1_)), where E_1_ and E_2_ are the background‐corrected mean mVenus fluorescence levels measured under full induction at the times t_1_ and t_2_, respectively. The expression rates were normalized by the corresponding cell density (OD). A τ plot (analogous to a Lineweaver‐Burk plot) shows the inverse of the mean transcription rate (estimated from the corresponding mVenus levels) against the inverse of the mean single‐cell RNAP concentrations. When changing the medium as described above, these two variables vary linearly with each other, allowing for the y‐intersect to be used as an estimate for the effective RNAP‐promoter engagement time [42].

### 5.2 RNA‐Seq Experiments and Analysis

#### 5.2.1 Sample Preparation

Cells from the overnight cultures were grown until the mid‐exponential phase (OD_600_ ≈ 0.3) in LB medium and were introduced to stresses (see above, considered as ‘time zero’). Samples were then collected 160 min after time zero, from three independent biological replicates. Control cultures were maintained in standard conditions (37°C and no antibiotics). For dual perturbation experiments, the same procedure was followed, and both stresses were applied at time zero.

After collecting the samples, 5 mL of the culture was immediately treated with a double volume (10 mL) of RNA protect bacteria reagent (Qiagen, Germany) for 5 min at room temperature to prevent RNA degradation. Next, the treated cells were pelleted and frozen at −80°C overnight. The next morning, total RNA was extracted using the RNeasy kit (Qiagen, Germany).

#### 5.2.2 Sequencing

Extracted RNA was treated twice with DNase (Turbo DNA‐free kit, Ambion, USA) and quantified using Qubit 2.0 Fluorometer RNA assay (Invitrogen, Carlsbad, CA, USA). The total RNA quality was determined using a 1% agarose gel stained with SYBR Safe (Invitrogen, Carlsbad, CA, USA), where RNA was detected using UV in a Chemidoc XRS imager (Bio‐Rad, USA). RNA integrity was measured by the Agilent 4200 TapeStation (Agilent Technologies, Palo Alto, CA, USA).

RNA library preparations, sequencing, and quality control analysis of sequenced data were conducted at GENEWIZ, Inc. (Leipzig, Germany). In detail, ribosomal RNA depletion was performed using Ribo‐Zero Gold Kits (Bacteria probe) (Illumina, San Diego, CA, USA), while the RNA sequencing library was prepared using the NEBNext Ultra RNA Library Prep Kit.

The sequencing libraries were multiplexed and clustered on one lane of a flow cell, which was loaded on an Illumina HiSeq 4000 instrument (after the thermal shift) or on an Illumina NovaSeq 6000 instrument (after adding the antibiotic). In both instruments, the samples were sequenced using a single‐index 2 × 150 Paired‐End (PE) configuration.

Image analysis and base calling were conducted by the HiSeq Control Software (Illumina HiSeq) and by the NovaSeq Control Software v1.7 (Illumina NovaSeq). The raw sequence data (.bcl files) were converted into “fastq” files and de‐multiplexed using Illumina bsl2fastq v.2.20. One mismatch was allowed in the index sequence identification.

#### 5.2.3 RNA‐Seq Data Analysis Pipeline

Shortly: (i) RNA sequencing reads were trimmed to remove possible adapter sequences and nucleotides with poor quality using Trimmomatic v.0.36. (ii) Trimmed reads were mapped to the reference genome, *E. coli* MG1655 (NC_000913.3), using the Bowtie2 aligner v.2.3.5.1, generating BAM files. (iii) Unique gene hit counts were calculated with featureCounts from the Rsubread R package (v.1.34.7). Genes with less than 5 counts in more than 3 samples and genes whose mean counts were smaller than 10 were removed from further analysis. (iv) The read counts were used for downstream differential expression analysis. The DESeq2 R package (v.1.24.0) [65] was used to calculate log_2_ of the fold changes (LFC) of RNA levels relative to the same control (standard growth temperature 37C at 160 min), and p‐values were calculated using Wald tests.

### 5.3 Principal Component Analysis

Global variance in gene expression was assessed by principal component analysis (PCA). Normalized LFC values were mean‐centered and scaled, prior to decomposition, using the prcomp function in R. Variance explained by each component was quantified from the eigenvalues, and scree plots were generated to summarize the contribution of the individual components.

### 5.4 Identification of Shifter Genes

To identify genes following temperature‐shift responses, but not antibiotic stresses when under dual stresses, we applied a residual‐based framework using linear regression. We compiled gene‐wise LFCs for temperature‐only (T), antibiotic‐only (AB), and the dual stress (TAB^(obs)^). Then, we retained the genes present in all three cases. To account for global offset and rescaling between conditions, we fitted two ordinary least‐squares (OLS) models:

$$\mathrm{TAB}\_\mathrm{mode}l^{\left(\mathrm{AB}\right)}=\alpha_{\mathrm{AB}}+m_{\mathrm{AB}}\cdot AB_{i}\;\mathrm{and}\;\mathrm{TAB}\_\mathrm{mode}l^{\left(T\right)}=\alpha_{T}+m_{T}\cdot T_{i}$$

For each gene *i*, we obtained fitted values TAB**^(AB)^** and TAB**^(T)^**, and computed absolute residuals (absolute differences between predicted values and actual values):

$$R_{\mathrm{AB}}\left(i\right)=|\mathrm{TA}B^{\left(\mathrm{obs}\right)}\left(i\right)-\mathrm{TAB}\_\mathrm{mode}l^{\left(\mathrm{AB}\right)}\left(i)\right|$$

$$R_{T}\left(i\right)=|\mathrm{TA}B^{\left(\mathrm{obs}\right)}\left(i\right)-\mathrm{TAB}\_\mathrm{mode}l^{\left(T\right)}\left(i)\right|$$

Genes were classified as thermal ‘shifters’ if their R**_Ab_** was within the 75th percentile (top quartile) while R**_T_** was below the 25th percentile (bottom quartile). This allowed us to identify the genes that shifted away from antibiotic influence but remained consistent with thermal shifting effects, while also filtering out false positives.

### 5.5 KEGG Pathway Analysis

Gene symbols were mapped to KEGG identifiers (eco) via the KEGG REST API, and associated pathways were retrieved. Over‐representation analysis was performed with clusterProfiler v4.4.1, using Benjamini‐Hochberg false discovery rate (FDR) correction (q < 0.05) to find significantly enriched pathways.

### 5.6 Heat Maps

Heatmaps of differentially expressed genes (|log_2_ fold change (LFC)| ≥ 2) were generated using the pheatmap R package (v1.0.12). A gene (rows) × condition (columns) matrix of LFCs was constructed, and the genes were hierarchically clustered using hierarchical clustering based on Euclidean distance and the complete linkage method in *pheatmap* (v1.0.12).

### 5.7 Gene Clusters

To classify genes according to their expression levels, the row dendrogram of the heatmaps was cut into 4 clusters, using the cutree function. For each cluster, the mean LFC across all conditions was calculated, and clusters were ranked in descending order of their average expression. Based on this ranking, clusters were assigned descriptive categories: ‘Strongly Upregulated’, ‘Upregulated’, ‘Downregulated’, and ‘Strongly Downregulated’. These categories were used to label genes for further downstream visualization and analysis.

### 5.8 Gene Ontology

Gene symbols were mapped to Entrez Gene IDs via org.EcK12.eg.db. GO enrichment was performed with clusterProfiler v.4.4.1 using the Biological Process ontology and *E. coli* K‐12 annotation database as reference. Significance was assessed with Benjamini‐Hochberg correction (*p* < 0.05). Enriched terms were visualized as dot plots, with the top categories reported per cluster.

### 5.9 Cellular ATP Levels

QUEEN‐2m‐expressing cells (a kind gift from Hiromi Imamura, Yamaguchi University) [36] were used to track ATP levels. We tracked ATP levels in a BioTek Synergy HTX Multi‐Mode Reader by exciting the solution at 400 nm and recording the emission at 513 nm. Afterward, the solution was re‐excited at 494 nm, and emission was recorded at 513 nm. The ratio between the emission intensities at the two excitation wavelengths (400ex/494ex) is used as a proxy for ATP levels [36].

### 5.10 Microscopy and Image Analysis

Cells were grown as described in Section 5.1. Cells were then fixed with 3.7% formaldehyde in phosphate‐buffered saline (PBS) for 30 min at room temperature, followed by washing with PBS twice to remove excess formaldehyde. The cell pellets were resuspended in phosphate‐buffered saline (PBS) with 2 µg/mL DAPI (4′,6‐diamidino‐2‐phenylindole) to stain the nucleoid. Cells were then incubated in the dark for 20 min, pelleted, and washed twice with PBS to remove excess DAPI. Cells were then re‐suspended in 100 µL of PBS.

Three microliters of cell suspension were placed on a 2% agarose gel pad and kept in between the round microscope slide and a coverslip. It took ∼5 min to move cells from the incubator to the microscope and start the observations. This interval includes the time for assembly of the microscope imaging chamber containing the slides and the cells.

Cells were visualized using a Nikon Eclipse *Ti‐E* inverted microscope with a C2‐plus confocal laser‐scanning system and a × 100 apochromatic TIRF oil DIC N2 objective (1.49 numerical aperture). For phase‐contrast imaging, we used a DS‐Fi2‐U3 camera (Emulated Mono) paired with the same objective. Phase‐contrast and confocal images were taken simultaneously.

YFP expression levels were visualized by a 488 nm laser and a 514/30 emission filter, while DAPI‐stained nucleoids were visualized by a 405 nm laser and a 447/60 emission filter. Cell borders were obtained from the phase‐contrast images.

Raw TIFF images were processed using Fiji/ImageJ (v 1.54 g). For each non‐overlapping field of view, DAPI and enhanced green fluorescent protein (EGFP) channels were converted into 8‐bit grayscale. Nucleoids were segmented from the DAPI channel by manual thresholding, applied separately for each condition, and, where necessary, morphological operations (watershed, fill holes) were applied to refine segmentation. Binary masks were generated to define regions of interest (ROI), respectively, and were used to quantify mean fluorescence intensity and area in both DAPI and EGFP channels. Particle detection was restricted to objects larger than 100 pixels.

### 5.11 Colocalization

Colocalization between DAPI (nucleoid) and GyrA‐YFP or RpoB‐YFP was evaluated using the ‘Colocalization Threshold plugin’ in Fiji. This plugin calculates the Manders’ overlap coefficients (M_1_, M_2_), which quantify the fraction of the two fluorophores’ signal intensities that spatially overlap.

### 5.12 Flow‐Cytometry

We measured cells’ fluorescence using an ACEA NovoCyte Flow Cytometer (ACEA Biosciences Inc., San Diego, USA). Cells were diluted (1:10000) into 1 mL of phosphate‐buffered saline (PBS) solution, vortexed for 10 s. In each condition, 3 biological replicates were measured. In each replicate, we collected data from 50000 cells. The flow rate was set to 12 µL/min. The data were collected by Novo Express software (ACEA Biosciences Inc.).

For detecting YFP, we used a blue laser (488 nm) for excitation and the fluorescein isothiocyanate detection channel (FITC‐H) (530/30 nm filter) for emission, with a core diameter of 7.7 µm and a PMT voltage of 600.

The lower bound for the detection threshold in FSC‐H was set to 5000 to remove interference from particles. We also removed the top 1% highest FITC‐H values. To remove abnormal cells, we used Tukey fences. Finally, we searched for additional abnormal measurements at the single‐cell level, but we did not find it.

For measuring Nile red efflux, cells were grown in M9 medium as described in Section 5.1 to an OD_600_ = 0.3 (0 min). Cultures were then subjected to thermal and/or ofloxacin stress conditions and incubated for 140 min. Cells were harvested by centrifugation (4,000 × *g*, 5 min) and resuspended in temperature‐equilibrated, divalent‐cation‐free 1 × PBS supplemented with 0.4% glucose and 1.0 1.0 mM ethylenediaminetetraacetic acid (EDTA). The suspensions were then incubated for 10 min at the respective temperatures. Ofloxacin was re‐supplemented to the buffer for the corresponding antibiotic‐treated condition. For efflux measurements, Nile Red was added to a final concentration of 7.5 µm. To establish efflux‐inhibited controls, 10 µm carbonyl cyanide *m*‐chlorophenyl hydrazone (CCCP) was added together with Nile Red to the corresponding control suspensions. Cells were subsequently incubated in the dark for 10 min at the respective treatment temperatures before flow‐cytometric analysis (leading to a total time of 160 min after first introducing the stress).

Following this, efflux activity was expressed as the reduction in the percentage of mean Nile Red fluorescence relative to the corresponding CCCP‐inhibited control, which is calculated as ([F_cccp_‐F_sample_]/F_cccp_) × 100, where F is fluorescence intensity. Higher values indicate greater Nile Red efflux.

### 5.13 Molecular Docking Experiments

Initial Molecular docking was performed using AutoDock 4.2. Structural models included *E. coli* DNA Gyrase (GyrA_2_GyrB_2_ heterotetramer, PDB: 6rku) and *E. coli* topoisomerase IV (ParC, PDB: 1zvu). Both contain conserved quinolone‐sensitive catalytic sites [16, 53]. Prior to docking, we assigned Kollman charges to protein atoms and Gasteiger charges to ligand atoms. Docking was performed keeping proteins rigid and ligands flexible. A docking grid box (50 × 60 × 80 Å, spacing 0.375 Å) was centered on the canonical binding site of the GyrA and ParC subunits [16] of Gyrase and topoisomerase IV, respectively. The conformational search used a Lamarckian Genetic Algorithm. The lowest‐energy pose was selected as the representative docking conformation for downstream molecular dynamics simulations. All docked complexes were visually analyzed using PyMOL (The PyMOL Molecular Graphics System, Version 3.0, Schrödinger, LLC).

### 5.14 Molecular Dynamics Simulations

All‐atom MD simulations were performed using GROMACS 2019.2 [50]. For *E. coli*, we simulated both the docked fluoroquinolone‐protein complexes as well as RNAP bound to rifampicin using the available co‐crystal structure (α_2_ββ′ core with σ factor, PDB: 4kmu). For other bacterial species, we used available protein‐ligand co‐crystal structures as follows: *Klebsiella pneumoniae* (Topoisomerase IV bound to compound 36, a delafloxacin derivative, PDB: 6WAA), *Pseudomonas aeruginosa* (DNA Gyrase bound to novobiocin, PDB: 7PTF), *Staphylococcus aureus* (DNA Gyrase bound to moxifloxacin, PDB: 5CDQ), *Streptococcus pneumoniae* (Topoisomerase IV bound to delafloxacin, PDB: 8C41), *Mycobacterium tuberculosis* (RNAP bound to rifampin, PDB: 5UH6), and *Mycobacterium smegmatis* (RNAP bound to rifampicin, PDB: 6CCV).

For all simulations, the CHARMM36m all‐atom protein force field was used [66], with systems solvated in an explicit TIP3P water model. Ligand parameters were generated with the CGenFF online server and converted to the GROMACS format [67]. Each protein‐ligand complex was solvated in a triclinic periodic box, neutralized with counterions, and energy‐minimized by the steepest descent algorithm [50].

System equilibration followed a standard two‐phase protocol: NVT (constant number, volume, and temperature) followed by NPT (constant number, pressure, and temperature) ensembles, with positional restraints on heavy atoms, using the v‐rescale thermostat [68] and the Parrinello‐Rahman barostat [69]. In all cases, simulations were performed at the target condition by setting the thermostat reference temperature to 288 K (cold), 310 K (standard), or 315 K (heat), respectively. To further resolve temperature‐dependent changes in the *E. coli* Gyrase‐norfloxacin and Topoisomerase IV‐norfloxacin complexes, additional simulations were performed at intermediate temperatures of 298 K (25°C), 307 K (34°C), and 313 K (40°C), using otherwise identical simulation parameters. Long‐range electrostatics were evaluated with particle‐mesh Ewald (PME) [70]. Finally, short‐range Coulomb and Lennard‐Jones interactions used CHARMM‐consistent real‐space cutoffs. All bonds to Hydrogen were constrained with LINCS [71]. Production trajectories were run for 30 nanoseconds per system, saving coordinates every 10 picoseconds.

Post‐simulation analysis was performed using the GROMACS toolkit. Trajectories were reimaged under periodic boundary conditions. Binding stability was quantified via protein‐ligand hydrogen bonds. Finally, short‐range interaction energies were decomposed into Coulombic and van der Waals contributions.

### 5.15 Gene Conservation Analysis

We used the rentrez R package to assess the conservation of *E. coli* genes across bacterial species. We first retrieved each gene's Entrez ID. Homologs were then identified in batches using gene‐specific queries in the NCBI Gene database. Gene identifiers from all search batches were merged, and species annotations were extracted using entrez_summary. Only genes with 5 or more homologs across species were retained for downstream conservation analysis.

Publicly available transcriptomic datasets containing temperature exposures comparable to our cold‐ and heat‐shock conditions were obtained from the NCBI databases. We did not identify comparable datasets with combined temperature and antibiotic exposure. Thus, the analysis was restricted to temperature‐only responses. When only raw RNA‐seq counts were available, differential expression was calculated using DESeq2 as described in Methods Section 5.2

Functional orthologs of *E. coli* genes were identified using KEGG Orthology. Organism‐specific gene‐to‐KO associations were retrieved through the KEGG REST API and linked to the locus tags, NCBI Gene identifiers, and gene symbols reported in each transcriptomic dataset. The complete list of examined organisms, together with their LFC and NCBI gene identifiers, is provided as a Supplementary File.

For each evaluable ortholog, the sign of the temperature‐induced LFC was compared with that of the corresponding *E. coli* gene. Responses were classified as concordant when both genes changed in the same direction and discordant when in opposite directions. Genes without an identifiable kegg ortholog (KO) assignment or an available expression measurement were classified as unavailable and excluded.

For each species, we calculated a concordance score by dividing the number of evaluable orthologs that changed in the same direction as their corresponding *E. coli* genes by the total number of evaluable orthologs in that species. This was calculated separately for the shifter cohort and the remaining temperature‐responsive genes as follows:

C*_s_* = No. evaluable orthologs concordant with *E. coli* / No. evaluable orthologs in species *s*

We also calculated a gene‐level concordance score for each *E. coli* shifter gene. This was defined as the number of evaluable species in which the corresponding ortholog changed in the same direction as the *E. coli* gene, divided by the total number of species in which that gene could be evaluated:

C_g_ = No. evaluable species showing concordant response for gene *g* / No. species in which *g* was evaluated.

### 5.16 Temperature‐Antibiotic State‐Space Analysis

For each reporter and condition, the mean FITC‐H fluorescence was calculated from single‐cell fluorescence measurements, after removing outliers using Tukey fences. Mean fluorescence was normalized to the untreated 37°C condition and expressed as a LFC, similarly to RNA‐seq calculations.

Then, the response of each combined temperature‐ofloxacin condition was compared with two corresponding single‐perturbation responses: temperature‐only response measured at the same temperature without ofloxacin and AB‐only response measured at 37°C. The absolute distances, *d*, between LFC values with changing T and changing AB (A) were calculated as:

$$dT=\left|LFC_{\left(T,A\right)}-LFC_{\left(T\right)}\right|$$

$$dAB=\left|LFC_{\left(T,A\right)}-LFC_{\left(opt,A\right)}\right|$$

A dominance score, *D*, was then defined as:

D=dAB−dT

Positive values indicated that the combined response was closer to the temperature‐only response and was therefore classified as temperature‐like. Negative values indicated that it was closer to the antibiotic‐only response and was classified as antibiotic‐like. A value of zero indicated equal distance from both responses. Further, for each combined condition, the percentage of temperature‐like genes, *Pt*, was calculated as:

$$Pt\;\;\left(\%\right)=100\times N\left(D>0\right)/N_{reporter}$$

Gene‐specific dominance scores are presented as heatmaps. The overall percentage of temperature‐like reporters was displayed across the measured temperature‐ofloxacin state space. For visualization of the overall response landscape, values between the measured conditions were interpolated using spline interpolation and constrained to the range of 0 ‐ 100%. The overlaid points represent the measured conditions, whereas the continuous surface represents the interpolation between these measurements.

### 5.17 Random‐Forest Classification and Feature Attribution

We identified which regulatory features most likely influence the dual‐stress transcriptomes using random forest (RF) classifiers [72]. Genes were included if their ∣LFC∣>1 and corresponding *p* < 0.05. For each thermal shift, all four datasets (from each antibiotic and their absence) were merged into one dataset, and 34 curated binary features were considered. All features were obtained from RegulonDB [55].

These features were encoded for each differently expressed gene (DEG) in binary form, as *X_f_* ∈ {0,1} (e.g., X_σ38_ = 1 if regulated by σ^38^). LFCs were also binarized into labels Y ∈ {0,1} (downregulated = 0, upregulated = 1).

After filtering, the analysis included 7993 and 5215 DEGs for cold and heat shock conditions, respectively. Class proportions were balanced and, therefore, no class weighting or resampling was required.

RF classifiers were trained separately for cold and heat shock with 400 trees, minimum leaf size of 10, random feature subsampling at splits, and Gini impurity as the split criterion. Model performance was estimated from Out‐of‐bag (OOB) error curves as a function of the number of trees. Feature importance was computed as OOB permuted predictor delta error. For interpretability, the direction of association for each top feature, ***f*,** was summarized by:

$$\Delta_{f}=P\left(Y=1\left|X_{f}=1\right.\right)-P\left(Y=1\left|X_{f}=0\right.\right)$$

where P(Y = 1∣X_f_ = 1) and P(Y = 1∣X_f_ = 0) denote the probability of up‐regulation when feature f is present or absent, respectively. Meanwhile, if *Δ_f_* > 0 indicates a higher likelihood of up‐regulation when feature *f* is present.

## Author contributions

**R.J. and A.S.R**. conceived the study. **R.J**. designed the experiments, assisted by **A.S.R. A.M.A**. performed all flow‐cytometry, microscopy, and spectrophotometry. **V.C. and S.D**. conducted the RNA‐seq experiments. **R.J**. executed the computational simulations, data analyses, and results figures. **R.J**. interpreted most of the data, with contributions from **A.S.R. and A.M.A. R.J**. wrote the original draft. **A.S.R**. revised and edited it. **P.R**. performed all measurements related to RNAP engagement times. All co‐authors revised it. **A.M.A**. created the illustrative figure, assisted by **A.S.R. and R.J. V.C., and S.D**. contributed equally and shares the right to be listed first in author order.

## Funding

This work was supported by the Sigrid Juséliuksen Säätiö (250213 and 260224 to A.S.R.), Suomalainen Tiedeakatemia (R.J. and S.D.), Opetushallitus [TM‐24‐12142] to A.M.A., Tampere University Graduate Program (V.C. and A.M.A.). The funders had no role in the study design, data collection and analysis, decision to publish, or preparation of the manuscript.

## Conflicts of Interest

The authors declare no conflicts of interest.

## Supporting information




**Supporting File**: advs77965‐sup‐0001‐SuppMat.docx.

## Data Availability

The RNA‐seq data generated for this study are available in EMBL‐EBI (https://www.ebi.ac.uk/biostudies/ArrayExpress/studies/E‐MTAB‐15747?key = 4a3e37f0‐ea3e‐4f46‐af08‐725200494bb0). Another data package was deposited in Zenodo (https://zenodo.org/records/17288155) with all source code along with flow cytometry, microscopy, and spectrophotometry data.
