# Supplementary material for: Thermal Shifts Dominate Single‐Gene Responses to Transcription‐Targeting Antibiotics
Source: Adv Sci (Weinh). 2026 Sep 27:e77965. Online ahead of print. doi: 10.1002/advs.77965 (PMC13616319; doi:10.1002/advs.77965)
Supplement: Supplementary file 1 [file ADVS-9999-e77965-s001.docx]

**Supplementary Information**

**Thermal Shifts Dominate Single-Gene Responses to Transcription-Targeting Antibiotics**

**Rahul Jagadeesan et al**

**Supplementary figure 1: Overlap of differentially expressed genes (DEGs) under antibiotic, thermal shift, and dual stresses.** (**A**) Antibiotics: norfloxacin (Nor), ofloxacin (Ofl), and rifampicin (Rif). Venn diagrams show upregulated (left) and downregulated (right) genes for each comparison. (**B**) Thermal shifts: cold shock (CS) and heat shock (HS). (**C**) CS vs antibiotics. (**D**) HS vs antibiotics. (**E**) CS vs dual (CS + antibiotics). (**F**) HS vs dual (HS + antibiotics). Only genes with |log₂ fold-change| ≥ 1 and *p* < 0.05 were classified as differentially expressed.

**Supplementary Figure 2: Scatter plots between antibiotic responses at optimal temperature.** Log₂ fold-change (LFC) under (**A**) ofloxacin (Ofl) vs. norfloxacin (Nor), (**B**) rifampicin (Rif) vs. norfloxacin, and (**C**) rifampicin vs. ofloxacin. Measured at the standard growth temperature (37 °C). Each dot represents a gene. The green line shows the best-fit linear regression, with (small) shaded 95% confidence interval. The coefficients of determination (R²) for statistically significant correlations (*p* < 0.05) are shown in each panel. Data were z-score normalized before plotting.

**Supplementary Figure 3. Scatter plots cold- and heat-shock induced transcriptional responses.** Log₂ fold-change (LFC) under cold shock(CS) vs heat shock (HS). Each dot represents a gene. The green line shows the best-fit linear regression, with (small) shaded 95% confidence interval. Also shown is the coefficient of determination (R²). Data were z-score normalized before plotting.

**Supplementary Figure 4. Scatter plots between antibiotic and cold- and heat-shock induced transcriptional responses.** Log₂ fold-change (LFC) under antibiotics at standard growth temperature (37 °C) versus cold shock (top) or heat shock (bottom) responses. Each dot represents a gene. The green line shows the best-fit linear regression, with (small) shaded 95% confidence interval. The coefficients of determination (R²) for statistically significant correlations (*p* < 0.05) are shown in each panel. Data were z-score normalized before plotting.

**Supplementary Figure 5. Scree plots from principal component analysis (PCA). Percentages of explained variance by the principal components (PCs) are shown.** (**A**) PCA performed for cold-shock and cold-shock + antibiotic conditions, respectively. (**B**) PCA performed for heat-shock and dual heat-shock and antibiotic conditions. Components other than PC1 contribute only marginally (<15%), indicating that most of the variance in each dataset is captured by the first principal component.

**Supplementary Figure 6: Scatter plots between responses to antibiotics with and without thermal shifts.** Log₂ fold-changes (LFC) to rifampicin versus norfloxacin or ofloxacin under (**A–B**) optimal temperature, (**C–D**) cold shock, and (**E–F**) heat shock. Each dot represents a gene. The green line shows the best-fit linear regression, with (small) shaded 95% confidence interval. The coefficients of determination (R²) for statistically significant correlations (*p* < 0.05) are shown in each panel. Data were z-score normalized before plotting.

**Supplementary Figure 7**. **Venn diagrams of shifter genes under dual stress.** (**A**) Heat shock with antibiotics (**B**) Cold shock with antibiotics. Shifter genes are those whose dual stress response diverges from antibiotic responses but converge with thermal shift responses (**Methods 4.4**).

**Supplementary Figure 8.** **KEGG pathway enrichment of temperature-specific shifter genes**. KEGG pathway enrichment analysis of (A) cold-shock-specific and (B) heat-shock-specific shifter genes. Only significantly enriched pathways are shown. The Rich factor represents the proportion of genes assigned to a given KEGG pathway that were present in the corresponding shifter-gene cohort, with higher values indicating strong enrichment. Bubble size is proportional to the number of shifter genes associated with each pathway, with the corresponding gene count indicated inside each bubble. Red bubbles denote metabolism-related pathways, whereas grey bubbles represent other enriched pathways.

**Supplementary Figure 9**: **Transcriptomic clustering under cold and dual stress involving cold shock.** Heatmap of differentially expressed genes (DEGs) under cold shock (CS) alone and in combination with antibiotics (norfloxacin (Nor), ofloxacin (Ofl), rifampicin (Rif)). Genes were clustered by hierarchical clustering of their log₂ fold-change values into four categories: Strongly upregulated, Upregulated, Downregulated, and Strongly downregulated. (**Methods 4.6 & 4.7**).

**Supplementary Figure 10**: **Functional enrichment under cold and dual stress involving cold shock.** Gene Ontology (GO) enrichment analysis of genes classified as strongly upregulated. Dot size represents the number of genes per GO term, while their color represents the adjusted p-values. Only Genes with ≥ 5 hits and GO annotations were included (Methods 4.7 & 4.8).

**Supplementary Figure 11**: **Functional enrichment under cold and dual stress involving cold shock.** Gene Ontology (GO) enrichment analysis of genes classified as strongly downregulated genes. Dot size represents the number of genes per GO term, while their color represents the adjusted p-values. Only Genes with ≥ 5 hits and GO annotations were included (Methods 4.7 & 4.8).

**Supplementary Figure 12**: **Transcriptomic clustering under heat and dual stress involving heat shock.** Heatmap of differentially expressed genes (DEGs) under heat shock (HS) alone and in combination with antibiotics (norfloxacin (Nor), ofloxacin (Ofl), rifampicin (Rif)). Genes were clustered by hierarchical clustering of their log₂ fold-change values into four categories: strongly upregulated, upregulated, downregulated, and strongly downregulated. (Methods 4.6 & 4.7).

**Supplementary Figure 13**: **Functional enrichment under heat and dual stress involving heat shock.** Gene Ontology (GO) enrichment analysis of genes classified as strongly upregulated. Dot size represents the number of genes per GO term, while their color represents the adjusted p-values. Only Genes with ≥ 5 hits and GO annotations were included (Methods 4.7 & 4.8).

**Supplementary Figure 14**: **Functional enrichment under heat and dual stress involving heat shock.** Gene Ontology (GO) enrichment analysis of genes classified as strongly downregulated genes. Dot size represents the number of genes per GO term, while their color represents the adjusted p-values. Only Genes with ≥ 5 hits and GO annotations were included (Methods 4.7 & 4.8).

**Supplementary Figure 15. Representative microscopy images under Ofloxacin treated conditions.** Representative single-cell images showing the DAPI-stained nucleoid (blue), YFP-tagged protein signal (yellow), and the merged channels. White outlines indicate the cell boundaries derived from the corresponding phase-contrast images. The coloured boxes indicate the respective experimental conditions shown in each panel.

**Supplementary Figure 16. Temperature dependence of the RNAP–promoter engagement parameter.** Y-intercepts obtained from the τ-plot analysis at 15, 37, and 42 °C. For each biological replicate, the inverse OD-normalized mVenus expression rate was plotted against the inverse mean single-cell RNAP concentration and fitted by linear regression. Filled circles represent the Y-intercepts obtained from individual biological replicates. Open circles indicate the mean Y-intercept at each temperature, and error bars represent the standard error of the mean (SEM). The Y-intercept provides an estimate of the effective RNAP–promoter engagement time.

**Supplementary figure 17: Levels of multidrug efflux pump components under thermal shifts.** Bar plots show log₂ fold-changes of *acrA* and *acrB* levels measured by RNA-seq 160 min after starting individual and dual stresses. Differences were significant (adjusted p < 0.05) for all conditions except norfloxacin and ofloxacin.

**Supplementary Figure 18. Root mean square deviation (RMSD) analysis of antibiotic–target complexes.** Time-resolved RMSD profiles of (top row) DNA gyrase bound to norfloxacin, (middle row) RNA polymerase (RNAP) bound to rifampicin, and (bottom row) topoisomerase IV bound to norfloxacin under cold shock, optimal, and heat-shock conditions, respectively, during molecular dynamics simulations (30 ns). RMSD reflects the conformational stability of each antibiotic–target complex.

**Supplementary Figure 19. Interaction energy profiles of antibiotic–target complexes under different temperature conditions.** Time-resolved interaction energies (kcal/mol) between antibiotics and their primary targets (DNA gyrase and norfloxacin (top row), RNA polymerase and rifampicin (middle row), and topoisomerase IV and norfloxacin (bottom row) during 30 ns of molecular dynamics simulations under cold shock, optimal, and heat-shock conditions. Interaction energies were calculated as the sum of Lennard-Jones and Coulombic contributions, with more negative values indicating stronger binding affinities.

**Supplementary Figure 20: Time resolved hydrogen bound profiles between antibiotic–target interactions under thermal shifts across bacterial species.** Each panel shows the number of hydrogen bonds formed between the antibiotic and its target protein during 30-ns molecular dynamics simulations under cold-shock (15 °C, blue), optimal (37 °C, grey), and heat-shock (42 °C, orange) conditions**.**

**Supplementary Figure 21. Ofloxacin minimum inhibitory concentration (MIC) assay in E. coli K-12 MG1655**. Cells were exposed to increasing concentrations of ofloxacin, and growth was quantified by OD. Bars represent the mean OD from three biological replicates, and error bars indicate the standard error of the mean (SEM). The tested ofloxacin concentrations are indicated on the x-axis.

.

**Supplementary Figure 22. Temperature- and antibiotic-like responses across the temperature–ofloxacin parameter space**. Heatmaps show the responses of ten representative shifter-associated transcriptional reporters across five temperatures (15, 25, 30, 39, and 42 °C) and three ofloxacin concentrations (0.5, 0.75, and 1 µg/mL). For each condition, the combined response was classified according to whether it was closer to the corresponding temperature-only (T) or antibiotic-only (AB) response. The colour scale represents the dominance score, with positive values indicating greater similarity to the temperature-only response and negative values indicating greater similarity to the antibiotic-only response.

**Supplementary Figure 23. Temperature-dependent hydrogen-bond profiles of *E. coli* gyrase–norfloxacin and topoisomerase IV–norfloxacin complexes.** Time-resolved numbers of intermolecular hydrogen bonds measured during 30-ns molecular dynamics simulations across the indicated temperatures. The top row shows the gyrase–norfloxacin complex and the bottom row the topoisomerase IV–norfloxacin complex. Each vertical bar represents the number of hydrogen bonds detected at a given simulation frame, and the inset reports the mean ± standard deviation over the trajectory. For direct comparison with the main manuscript, the 15, 37, and 42 °C conditions shown in Figure 7A are reproduced here alongside the additional intermediate-temperature simulations.

**Supplementary Figure S24. Random forest classification of transcriptional responses under cold- and heat-shock.** (A–B) Out-of-bag (OOB) classification error as a function of the number of trees for cold (A) and heat-shock (B). (C-D) Corresponding confusion matrices showing the prediction accuracy for downregulated (Down) and upregulated (Up) genes under cold-shock (C) and heat-shock (D). Shown are the number of genes and the percentage correctly or incorrectly classified.

**Supplementary Tables:**

| Antibiotic Target and Temperature (°C) | µ (H-bonds) | σ (H-bonds) | Occupancy (time %) |
| --- | --- | --- | --- |
| Gyrase (15) | 1.38 | 0.024 | 89.31 |
| Gyrase (37) | 2.01 | 0.025 | 99.40 |
| Gyrase (42) | 0.42 | 0.021 | 31.86 |
| RNAp (15) | 3.26 | 0.025 | 100.00 |
| RNAp (37) | 4.13 | 0.033 | 100.00 |
| RNAp (42) | 3.69 | 0.028 | 100.00 |
| Topoisomerase IV (15) | 0.19 | 0.017 | 13.18 |
| Topoisomerase IV (37) | 1.93 | 0.023 | 97.70 |
| Topoisomerase IV (42) | 1.29 | 0.022 | 87.11 |

**Supplementary Table 1: Hydrogen bond statistics for antibiotic–target complexes estimated by molecular dynamics simulations (30 ns) in *E. coli***. Shown are the estimated average number of hydrogen bonds (µ), the standard error of the mean (SEM), and occupancy percentage, which is the proportion of simulation frames in which at least one hydrogen bond was detected.

| Conditions | Coulomb | Error (C) | Lennard-jones | Error (LJ) | Total Interaction energy (Kcal/ mol) | Total error |
| --- | --- | --- | --- | --- | --- | --- |
| Gyr 37 | -158.23 | 6.2 | -56.54 | 1.3 | -214.78 | 6.33 |
| Gyr 15 | -119.26 | 20 | -44.72 | 4.1 | -163.99 | 20.41 |
| Gyr 42 | -38.18 | 15 | -13.14 | 7.1 | -51.33 | 16.59 |
| Top 37 | -53.61 | 2.9 | -113.73 | 0.71 | -167.34 | 2.98 |
| Top 15 | -9.23 | 4.7 | -47.73 | 20 | -56.97 | 20.54 |
| Top 42 | -50.14 | 3.4 | -103.31 | 2.9 | -153.45 | 4.46 |
| RNAp 37 | -111.02 | 3.2 | -181.65 | 2 | -292.67 | 3.77 |
| RNAp 15 | -82.61 | 3.7 | -199.89 | 5.8 | -282.50 | 6.87 |
| RNAp 42 | -90.98 | 4 | -190.56 | 6.6 | -281.54 | 7.71 |

**Supplementary Table 2:** **Interaction energy components for antibiotic–target complexes estimated by molecular dynamics simulations (30 ns) in *E. coli*.** Shown are the Coulombic (electrostatic) and Lennard-Jones (van der Waals) contributions, their associated errors, and the resulting total interaction energy with propagated error, calculated using the error propagation method.

| Species, Antibiotic Target and Temperature (°C) | Occupancy (time %) |
| --- | --- |
| *K. pneumoniae*-Topo IV-FQ (15) | 1.89 |
| *K. pneumoniae*-Topo IV-FQ (37) | 80.81 |
| *K. pneumoniae*-Topo IV-FQ (42) | 28.47 |
| *M. smegmatis*-RNAp-Rif (15) | 95.90 |
| *M. smegmatis*-RNAp-Rif (37) | 96.70 |
| *M. smegmatis*-RNAp-Rif (42) | 80.01 |
| *M. tuberculosis*-RNAp-Rfm (15) | 99.70 |
| *M. tuberculosis*-RNAp-Rfm (37) | 100.00 |
| *M. tuberculosis*-RNAp-Rfm (42) | 99.30 |
| *P. aeruginosa*-Gyrase-Nov (15) | 100.00 |
| *P. aeruginosa*-Gyrase-Nov (37) | 100.00 |
| *P. aeruginosa*-Gyrase-Nov (42) | 99.90 |
| *S. aureus*-Gyrase-MFX (15) | 19.78 |
| *S. aureus*-Gyrase-MFX (37) | 76.92 |
| *S. aureus*-Gyrase-MFX (42) | 45.05 |
| *S. pneumoniae*-Topo IV-TE (15) | 60.13 |
| *S. pneumoniae*-Topo IV-TE (37) | 85.71 |
| *S. pneumoniae*-Topo IV-TE (42) | 72.32 |

**Supplementary Table 3:** **Hydrogen bond statistics for antibiotic–target complexes across multiple bacterial species estimated by molecular dynamics simulations (30 ns).** Shown is the occupancy time percentage, which represents the proportion of simulation frames in which at least one hydrogen bond was detected. Species and complexes shown are *Klebsiella pneumoniae* Topoisomerase IV–fluoroquinolone (FQ), *Mycobacterium smegmatis* RNA polymerase–rifampicin (Rif), *Mycobacterium tuberculosis* RNA polymerase–rifampicin (Rfm), *Pseudomonas aeruginosa* Gyrase–norfloxacin (Nov), *Staphylococcus aureus* Gyrase–moxifloxacin (MFX), and *Streptococcus pneumoniae* Topoisomerase IV–tetracycline (TE).

| Organism | Pearson correlation coefficient | p-value |
| --- | --- | --- |
| Leuconostoc gelidum | 0.44 | 0.03 |
| Paucilactobacillus oligofermentans | 0.47 | 0.01 |
| Lactococcus piscium | 0.39 | 0.00 |

**Supplementary Table 4:** **Correlation of orthologous gene expression responses between *E. coli* and psychrotrophic lactic acid bacteria (*Leuconostoc gelidum*, *Paucilactobacillus oligofermentans*, and *Lactococcus piscium*).** Shown are the Pearson correlations coefficients and their and their associated p-values. Data was obtained from published RNA-seq datasets that most closely matched our experimental setup (timing and temperature conditions).
